# Proteomic analysis and interactions network in leaves of mycorrhizal and nonmycorrhizal sorghum plants under water deficit

**DOI:** 10.7717/peerj.8991

**Published:** 2020-04-23

**Authors:** Víctor Olalde-Portugal, José Luis Cabrera-Ponce, Argel Gastelum-Arellanez, Armando Guerrero-Rangel, Robert Winkler, Silvia Valdés-Rodríguez

**Affiliations:** 1 Departamento de Biotecnología y Bioquímica, Centro de Investigación y de Estudios Avanzados del IPN-Unidad Irapuato, Irapuato, Guanajuato, México; 2 Departamento de Ingeniería Genética, Centro de Investigación y de Estudios Avanzados del IPN-Unidad Irapuato, Irapuato, Guanajuato, México; 3 Área de Medio Ambiente y Biotecnología, Cátedra CONACYT. Centro de Innovación Aplicada en Tecnologías Competitivas A.C. (CIATEC AC), León, Guanajuato, México

**Keywords:** Water deficit, Arbuscular mycorrhizal fungi, Protein–protein interaction, Gene network, Ribosomal proteins, ATP synthase, Signaling, Photosynthesis, Proteomic analysis, Sorghum

## Abstract

For understanding the water deficit stress mechanism in sorghum, we conducted a physiological and proteomic analysis in the leaves of *Sorghum bicolor* L. Moench (a drought tolerant crop model) of non-colonized and colonized plants with a consortium of arbuscular mycorrhizal fungi. Physiological results indicate that mycorrhizal fungi association enhances growth and photosynthesis in plants, under normal and water deficit conditions. 2D-electrophoresis profiles revealed 51 differentially accumulated proteins in response to water deficit, of which HPLC/MS successfully identified 49. Bioinformatics analysis of protein–protein interactions revealed the participation of different metabolic pathways in nonmycorrhizal compared to mycorrhizal sorghum plants under water deficit. In noninoculated plants, the altered proteins are related to protein synthesis and folding (50S ribosomal protein L1, 30S ribosomal protein S10, Nascent polypeptide-associated complex subunit alpha), coupled with multiple signal transduction pathways, guanine nucleotide-binding beta subunit (Rack1) and peptidyl-prolyl-cis-trans isomerase (ROC4). In contrast, in mycorrhizal plants, proteins related to energy metabolism (ATP synthase-24kDa, ATP synthase *β*), carbon metabolism (malate dehydrogenase, triosephosphate isomerase, sucrose-phosphatase), oxidative phosphorylation (mitochondrial-processing peptidase) and sulfur metabolism (thiosulfate/3-mercaptopyruvate sulfurtransferase) were found. Our results provide a set of proteins of different metabolic pathways involved in water deficit produced by sorghum plants alone or associated with a consortium of arbuscular mycorrhizal fungi isolated from the tropical rain forest Los Tuxtlas Veracruz, México.

## Introduction

Sorghum, *Sorghum bicolor* L. Moench, is a multipurpose C4 cereal crop, with high photosynthetic rate and productivity (Jagtap et al., 1998). It ranks the fifth economically important cereal crop worldwide (Food Agriculture Organization of the United Nations, FAO). Sorghum plays a vital role in sustainable farming, as it is particularly resilient to stress conditions caused by drought and erratic rainfall (Buchanan et al., 2005). Drought resistance is a complex trait, which depends on the action and interaction of different morphological, physiological, biochemical and genetic mechanisms (Mitra, 2001). Molecular breeding approaches through the identification of QTLs and marker-assisted selection in drought-tolerant sorghum genotypes have identified fifteen genes highly induced in response to drought stress (Abou-Elwafa & Shehzad, 2018). Clustering of these drought-responsive genes suggests a conserved evolutionary mechanism to promote the existence and maintenance of clusters, which could be implicated in a common metabolic pathway to produce a protein complex or serve as receptors in signaling pathways (Abou-Elwafa & Shehzad, 2018). Proteomics is a biotechnological approach for drought improvement; it is a tool for the identification of proteins involved in cellular processes and provides information on the amount of gene products, their isoforms, and which post-transcriptional modifications regulate protein activation (Aslam et al., 2017). Proteomic analysis of the adaptive response of sorghum to drought and subsequent recovery indicated that proteins related to energy balance, metabolism, and chaperones were the most apparent features to elucidate the differences between the drought-tolerant and sensitive plants (Jedmowski et al., 2014). In sorghum cell suspension subjected to osmotic stress with sorbitol, the secretion of glycosyl-hydrolases/glycosidases, cell wall modifying enzymes, proteases, and redox proteins was increased, suggesting that extracellular matrix proteins had a wide range of functions in adaptive drought-stress responses (Ngara et al., 2018). Arbuscular mycorrhizal (AM) fungi (subphylum Glomeromycotina (Spatafora et al., 2016) establish a biotrophic mutualistic association, called AM symbiosis with more than 80% of angiosperms and gymnosperms based on the current knowledge of Brundett & Tedersoo (2018). AM fungi consume plant photosynthates (Bago, Pfeffer & Shachar-Hill, 2000), and fatty acids to complete their life cycle (Keymer et al., 2017; Bravo et al., 2017). AM fungi improve the nutrition and water acquisition of plants, inducing gene expression of transporters of carbon, nitrogen, phosphorous, sulfate, potassium, and water exchanges (Wipf et al., 2019) and heavy metals (Tamayo et al., 2014; Dhawi, Datta & Ramakrishna, 2016). It also protects plants against fungal pathogens (Pozo & Azcón-Aguilar, 2007; Liu et al., 2007; Akhtar & Siddiqui, 2008) and other abiotic stress (Augé, 2001; Miransari et al., 2008; Lenoir, Fontaine & Sahraoui, 2016). Furthermore, it modulates plant growth and development through CLAVATA 3/embryo surrounding region related (CLE) proteins (LeMarquer, Bécard & Freidit, 2019). These proteins are plant specific peptidic hormones that act as mediators of cell-to-cell communication. CLE proteins have been associated to stem cell homeostasis of the shoot and root apical meristems (SAM and RAM, respectively) (Wang, Zhang & Wu, 2016). Arbuscular mycorrhizal (AM) fungi (*Funneliformis mossae*) improved performance of forage sorghum, prolonged lifespan, and significantly increased growth and protection against abiotic stress conditions (Sun, Shi & Ding, 2017). Sorghum grown in marginal soils and inoculated with four species of mycorrhizal fungi (*Rhizophagus aggregatus, G. etunicatum*, *Funelliformis mossae* and *Rhizophagus irregularis*), improved mineral nutrition (P, S, Fe, Ca, K, Cu, Zn) and activates a set of seven proteins (Dhawi, Datta & Ramakrishna, 2016; Dhawi, Datta & Ramakrishna, 2017). Recently, Symanczik et al. (2018), demonstrated that *Rhizophagus arabicus*, an arbuscular mycorrhizal fungus endemic of arid ecosystems transfers more efficiently nitrogen and phosphorous than *R. irregularis*, strain from temperate climates in sorghum plants under drought conditions. It also has been demonstrated that root inoculation with two or more species of AM fungi confers more benefits to plants than the addition of a single species (Chen et al., 2018). In the present work, we made a differential proteomic analysis of mycorrhizal and nonmycorrhizal sorghum plants under water deficit conditions based on the use of two-dimensional gel electrophoresis (2DE). Bioinformatics analysis with STRING (0.500) confidence revealed different metabolic pathways in nonmycorrhizal compared to mycorrhizal sorghum plants under water deficit stress. Nonmycorrhizal sorghum plants produced mainly proteins associated with ribosome biogenesis under water deficit conditions, while mycorrhizal plants produced proteins associated with energy metabolism, carbon metabolism, glycolysis/gluconeogenesis, oxidative phosphorylation, and photosynthetic carbon fixation.

## Materials & Methods

### Plant material

Seeds of *Sorghum bicolor* L. Moench cv. BJ-83 Caloro

### Evaluation of physiological parameters in sorghum plants

In the present work, we evaluated arbuscular mycorrhizal (AM) colonization and physiological parameters related to water deficit treatment, such as plant and soil water potential, and gas exchange measurements in nonmycorrhizal and mycorrhizal (fungal consortium) sorghum plants under well-watered (WW) and water deficit (WD) conditions.

### Water deficit treatment

The experiments followed a randomized complete block design. Sixty 2.3-L plastic pots (15/treatment) were seeded with *S. bicolor* L. Moench cv. BJ-83 Caloro with one seed per pot. The potting substrate was pasteurized (coarse sand-sandy loam 1:1). Thirty pots consisted in sorghum seeds inoculated with a culture of a fungal consortium of arbuscular mycorrhizae (AM) from the tropical rain forest Los Tuxtlas Veracruz, México. The consortium is integrated by *Septoglomus constrictum*, *Funneliformis geosporum*, *Rhizoglomuss fasciculatum*, *Glomus toruosum*, *Gigaspora margarita* and *Acaulospora scrobiculata*. Sorghum seeds were inoculated with an average of 120 spores per pot. Other 30 pots received non-AM culture, and they all were grown in a glasshouse with a temperature maintained at 25−30 °C/18−22 °C (day/night) and PPFD (Photosynthetic Photon Flux Density) 1000 µmol m^−2^ s^−1^, in Irapuato, Guanajuato, México. Plants were watered as needed before stress applications. Plants were irrigated with distilled water as required and fertilized weekly with 200 mL per pot using Long Ashton nutritive solution (Hewitt, 1966), supplemented with phosphorous. Nonmycorrhizal plants were fertilized with 44 mg/L of KH_2_PO_4_, while AM-plants with 22 mg/L KH_2_PO_4_, according to Cho et al. (2006). After 45 days, 30 pots -15 nonmycorrhizal (WD) and 15 mycorrhizal (WDM) plants- were subjected to water-deficit stress by a progressive decrease of water 65, 55, 40, 25, 13 and 0% of water retention capacity (WRC), during six days. The initial WRC soil was 16.77% and the level of water deficit was adjusted adding distilled water. Thirty pots (15 nonmycorrhizal (WW) and 15 mycorrhizal plants (WWM)) remained well watered. At day 7, the plants were collected from both water-stressed plants and continuously watered plants of nonmycorrhizal and mycorrhizal sorghum plants. The plants were subjected to different analyses as described below. For proteomic analysis, leaves for different plants were frozen in liquid nitrogen and stored at −70 °C for further analysis.

### Arbuscular mycorrhizal (AM) colonization

Root segments of four plants per treatment were scored for colonization of AM according to Phillips & Hayman (1970). Arbuscules and vesicles in the root cortex were recorded, values were expressed as percentages.

### Plant and soil water potential

Measurements of soil and leaves Ψ were made with Wescor Dew Point Microvoltmeter Model HR 33T (WESCOR, Inc, Logan, Utah, USA), before and after water deficit treatments at 45 days and 52 days after sowing, respectively. The evaluation was made in four plants in each treatment. For soil Ψ, gibbsite blocks connected to the microvoltmeter were set inside the pots 14 cm from top. Leaves Ψ was measured at 11:00 am in disks of the third youngest leaf in a C52 psychrometer chamber, which was equilibrated for 30 min before measuring with the Wescor Dew Point Microvoltmeter.

### Soil moisture

Soil moisture content was made according to Reynolds (1970). Soil core sample from the pots was taken with a cork borer. The soil was immediately weighed (FM, fresh mass) and then dried at 105 °C to determine the DM (dry mass) by gravimetric analysis.

### Gas exchange measurements

Gas exchange was determined on two fully developed leaves for four plants for each treatment using a LICOR-6200 Portable Photosynthesis System (LICOR, Inc., Lincoln, NE, USA). Measurements were made from 10:00 to 12:00 h under PPFD of 1,000 µmol m^−2^ s^−1^. The light was supplied by 400 W high sodium vapor pressure lamps (GE; Circleville, OH. USS, USA) filtered through 5 cm of water enclosed in a plexiglass box. Gas exchange measured included net photosynthetic rate (A), stomatal conductance (gs) and transpiration rate (E).

### Plant growth and biomass analysis

Plant height and stem diameter from four plants of each treatment was measured by using a precision straight edge and vernier caliper. The leaves and roots per plant were collected and dried in an oven at 80 °C for 24 h and weighted to record their dry biomass.

### Statistics

The values of the variables evaluated above were subjected to an analysis of variance (ANOVA), using the Tukey‘s test (*p* ≤ 0.05) for the cases of differences. The percentage values of mycorrhizal root colonization, arbuscules, and vesicles were analyzed by the LSD test. All the statistical analyzes were executed in the R 3.2.5 environment (R Core Team, 2018).

### Two-Dimensional Gel Electrophoresis (2DE)

Sorghum leave proteins were extracted using a phenol extraction method, according to Isaacson et al. (2006). Protein pellet was dissolved overnight in rehydration buffer (7 M urea, 2 M thiourea, 3% (*w*/*v*) 3-[3-Cholamidopropyl) dimethyl-ammonio] propanosulfonic acid CHAPS, 0.5% (*v*/*v*) IPG buffer (pH 4–7) and 20 mM DTT at room temperature. Protein concentration was quantified using BioRad Protein assay (BioRad, CA, USA) with BSA as the standard. Proteins were subjected to two-dimensional electrophoresis according to Görg, Drews & Weiss (2006), with minor modifications. Commercial immobilized pH gradient (IPG) strips (GE Healthcare Life Sciences, Uppsala, Sweden) of 18 cm in length and pH 4–7 were loaded with 1 mg of protein. Isoelectric focusing (IEF) was performed in an Ettan IPGphor III system (GE Healthcare, Bio-sciences AB, Uppsala, Sweden) at 22 °C. The second dimension was performed in a 12.5% SDS-PAGE and stained by Coomassie Brilliant Blue staining. Gels for four biological samples from each treatment were obtained. Gel images (300 dpi, 12-bit per inch) were scanned using an Image Scanner II UTA-1100 (Amersham BioScience, China) and were analyzed and compared using the Melanie 2D software version 7.0 (GeneBio, Geneva, Switzerland). Protein spots were quantified by their relative volume expressed as % relative volume, which represents the ratio of the volume of a single spot to the whole set of spots.

### Proteomic statistical analysis

Four biological samples for each experimental condition were analyzed. Statistical analysis was carried out in the R 3.2.5 environment (R Core Team, 2018). Raw data pre-processing was divided into three consecutive steps: missing value imputation, normalization, and transformation, according to Valledor & Jorrín (2011). Multivariate analysis was performed over the whole set of pre-processed data. As a first stage, standardized principal component analysis (PCA) was performed using the “ade4” package (Dray & Dufour, 2007). Subsequently, an independent component analysis (ICA) was applied to the PCA patterns, by means of the “fastICA” package (Marchini, Heaton & Ripley, 2013). Hierarchical clustering analysis (HCA) was also carried out on the PCA patterns, by means of the “FactoMineR” package and validated by bootstrap analysis using the package “pvclust” to calculate the approximately unbiased probability value, AU (Husson et al., 2015). Spots with the highest absolute loadings on PCA-ICA and the lowest *p*-values on PCA-HCA were selected and identified by mass spectrometry. A heat map was constructed with all the relevant information obtained. A univariate analysis was performed for assessing significant differential abundances of particular spots between the four experimental conditions, and differences were considered statistically significant at *p* ≤ 0.05.

### Protein Identification

Protein spots with significant changes were manually excised from the gel for protein identification. Proteins were digested with sequencing grade trypsin (Promega, article number V511A, Madison, Wisconsin, USA) as described previously by Shevchenko et al. (2006). Tryptic peptides were extracted, lyophilized, and analyzed by Mass spectrometry (MS). MS analysis was performed by a nanoacquity LC system (Waters, Milford, MA, USA) coupled to a linear ion trap (LTQ) Velos mass spectrometer (Thermo Fisher Scientific, Bremen, Germany) equipped with a nanoelectrospray ion source. The spectrometer was operated in positive mode, and a full scan was acquired over mass to charge ratio (*m*/*z*) 400–2,000 and automatically switching to MS/MS on most intense peaks of charges 2^+^ and 3^+^. Data were extracted by Proteome Discoverer version 1.2 (Thermo Fisher Scientific Inc., USA) and searched by SEQUEST search engine (Thermo Fisher Scientific Inc., USA) against a Sorbidraft database (34,026 entries). Searches were executed with the following parameters: 2 Da parent MS ion window, 1 Da MS/MS ion window, and two missed cleavages allowed. The iodoacetamide derivative of cysteine (carbamidomethyl cysteine) was specified in Sequest as a fixed modification, oxidation of methionine as a variable modification. A decoy database search with false discovery rates < 0.05 was used. Theoretical Mr and pI of the identified proteins were predicted using http://web.expasy.org/compute_pi (Gasteiger et al., 2003). Sequences of identified proteins were used to search homologous by the BLAST protein algorithm against the GenBank non-redundant protein database (Viridiplantae) (https://blast.ncbi.nlm.nih.gov/Blast.cgi) (NCBI Resource Coordinators, 2017). The subcellular location of identified proteins was predicted using the public program WolfPsort (https://www.genscript.com/wolf-psort.html) (Horton et al., 2007).

### Interaction analysis of proteins involved in WD and WDM sorghum plants

A gene network with (0.500) confidence was performed with STRING (v11.0, http://string-db.org) (Szklarczyk et al., 2019), based on *Sorghum bicolor* genes. Gene identifier (Id) was made according to Uniprot (http://www.uniprot.org) (UniProt Consortium, 2014), NCBI (http://www.ncbi.nlm.nih.gov) (NCBI Resource Coordinators, 2017). Sol genomics network (https://solgenomics.net) (Bombarely et al., 2010) and Phytozome (https://phytozone.jgi.doe.gov) database (Mitros et al., 2012).

## Results

### AM colonization

AM colonization was not influenced by water deficit. Well-watered mycorrhizal (WWM) sorghum plants showed 78.90 ± 8.50% root colonization, 61.60 ± 17.40% vesicles and 26.70 ± 5.15% arbuscules formation, and these values changed to 76.70 ± 8.10%, 40.20 ± 6.80% and 34.40 ± 5.50%, respectively, under water deficit (WDM), but they were not statically significant by the LSD test (*p* ≤ 0.05). Additionally, we confirmed that noninoculated sorghum plants were not colonized (Table 1).

**Table 1:** Percentage of mycorrhizal colonization, vesicles and arbuscules formation in sorghum roots under well-watered (WW) and water deficit (WD) conditions. Values are means ± SD of root segment observations from represents no significant differences among treatments according LSD test (*p* ≤ 0.05). * WW and WD represent well-watered and water deficit in nonmycorrhizal plants, while **WWM and WDM correspond to well-watered and water deficit mycorrhizal plants, respectively.

**Treatments**	**Colonization** **(%)**	**Vesicles** **(%)**	**Arbuscules** **(%)**
*WW	0	0	0
**WWM	78.90 ± 8.50 a	61.60 ± 17.40 a	26.70 ± 5.15 a
*WD	0	0	0
**WDM	76.70 ± 8.10 a	40.20 ± 6.80 a	34.40 ± 5.50 a

### Dry mass and morphometric parameters

Mycorrhizal colonization stimulated sorghum plant growth under well-watered conditions WWM, showing higher foliage and root dry mass than nonmycorrhizal WW plants (Table 2). However, at water deficit conditions, WDM and WD plants reduced dry mass foliage by 12.89% and 20%, compared to well-watered controls, respectively. Despite this decrease, WDM plants showed higher height plants than did WW and WD plants (Table 2).

**Table 2:** Dry mass and morphometric parameters of nonmycorrhizal and mycorrhizal (fungal consortium) sorghum plants under well-watered (WW, WWM) and water deficit (WD and WDM) conditions. These values represent the means ± SD of four plants for each treatment. The same letter in the columns represents no significant differences among treatments according to Tukeys test (*p* ≤ 0.05). * WW and WD represent well-watered and water deficit in nonmycorrhizal plants, while **WWM and WDM correspond to well-watered and water deficit mycorrhizal plants, respectively. ^c^DMR/DMF means dry mass root/dry mass foliage.

**Treatments**	**Dry mass foliage** **(g)**	**Dry mass root** **(g)**	^c^**DMR/DMF**	**Diameter** **stem** **(mm)**	**Height** **Plant** **(cm)**
*WW	7.32 ± 0.20 b	1.56 ± 0.38 b	0.21 ± 0.04 b	8.38 ± 0.44 b	84.36 ± 0.16 c
**WWM	8.30 ± 0.20 a	2.96 ± 0.42 a	0.36 ± 0.05 a	10.14 ± 0.26 a	96.42 ± 2.96 a
*WD	5.85 ± 0.65 c	1.46 ± 0.38 b	0.25 ± 0.05 ab	7.85 ± 0.34 b	80.70 ± 1.60 c
**WDM	7.23 ± 0.52 b	2.48 ± 0.37 a	0.34 ± 0.06 a	8.0 3 ± 0.16 b	90.70 ± 1.20 b

### Soil moisture, water potentials (Ψ), CO_**2**_ assimilation and stomatal conductance

Under our stress conditions, a significant reduction in the soil water potential of both WDM and WD plants was observed compared with that of the corresponding well-watered conditions (Table 3). However, mycorrhizal sorghum plants were able to adjust this stress and maintained their leaf water potential (-0.28.70 ± 0.008 MPa) very similar to that of nonmycorrhizal WW plants (−0.27 ± 0.014 MPa). Additionally, WDM plants showed a higher stomatal conductance (112 ± 3.6 mmol m^−2^ s^−1^) than WD plants (89 ± 4.24 mmol m^−2^ s^−1^). Rate of CO_2_ assimilation was higher in WWM (33.85 ± 2.3 µmol m^−2^ s^−1^) than in WW (28.70 ± 1.16 µmol m^−2^ s^−1^) sorghum plants, and these values decreased less in response to water deficit in WDM (26.70 ± 0.79 µmol m^−2^ s^−1^) than in WD plants (16.88 ± 1.8 µmol m^−2^ s^−1^) (Table 3). These results indicate that mycorrhizal fungal associations enhance photosynthesis in plants under normal and water deficit conditions.

**Table 3:** Soil moisture, water potentials (Ψ), CO_2_ assimilation and stomatal conductance of sorghum plants under well-watered (WW, WWM) and water deficit (WD, WDM) conditions. These values represent the means ± SD of four biological samples for each treatment. The same letter in the columns represents no significant differences among treatments according to Tukey’s test (*p* ≤ 0.05). * WW and WD represent well-watered and water deficit nonmycorrhizal plants, while **WWM and WDM correspond to well-watered and water deficit mycorrhizal plants, respectively.

**Treatments**	**Soil moisture** (%)	**Ψ soil** **(MPa)**	**Ψ leaves** **(MPa)**	**CO**_**2**_**assimilation** **(µmol m**^−2^**s**^−1^**)**	**Stomatal conductance** **(mmol m**^−2^**s**^−1^)
*WW	23.0 ± 2.60 a	−0.20 ± 0.02 a	−0.27 ± 0.014 b	28.70 ± 1.16 b	193 ± 1.16 a
**WWM	19.0 ± 1.83 b	−0.30 ± 0.07 a	−0.17 ± 0.004 a	33.85 ± 2.3 a	125 ± 5.23 b
*WD	4.8 ± 0.28 c	−0.73 ± 0.38 b	−0.42 ± 0.016 c	16.88 ± 1.8ubrk c	89 ± 4.24 d
**WDM	4.0 ± 0.15 c	−0.81 ± 0.03 b	−0.28 ± 0.008 b	26.70 ± 0.79 b	112 ± 3.60 c

### Proteomic analysis

Differential proteomic analysis of *S. bicolor* leaves from plants subjected to water deficit and well-watered conditions, and in symbiosis with a consortium of arbuscular mycorrhizal was performed by 2DE electrophoretic profile. Four treatments were evaluated: well-watered nonmycorrhizal plants (WW), water deficit nonmycorrhizal plants (WD), well-watered mycorrhizal plants (WWM), and water deficit mycorrhizal plants (WDM). Protein electrophoretic profile displayed about 450 protein spots under different treatments. Representative gels (pH 4–7) of the distribution of proteins under the different treatments are shown (Fig. S1).

### Proteomic statistical analysis

Gels for four biological samples from each treatment were analyzed. The mean coefficient of variation (CV) of 25.7% was calculated from the raw data obtained from the image analysis. This value, which is common in raw proteomic data, was reduced to 8.5% when the pre-processing stage was applied without disturbing the original structure of the dataset. To evaluate differences in protein profiles among treatments, ICA and HCA were carried out on the PCA patterns obtained from the dataset. When plotting the results of the ICA (IC’s 1 and 2), an effective separation of samples into their original groups was observed, with IC1 showing a clear separation between water deficit (WD, WDM) and well watering (WW, WWM) plants. On the other hand, IC2 showed differences between plants inoculated with arbuscular mycorrhizal fungi (WDM, WWM) and those that were not inoculated (WD, WW), representing the plant-mycorrhiza association effects over the protein profiles (Fig. S2A). HCA results showed that replicates of each treatment clustered together, revealing four main groups strongly supported by the data (Fig. S2B).

**Figure 1:**
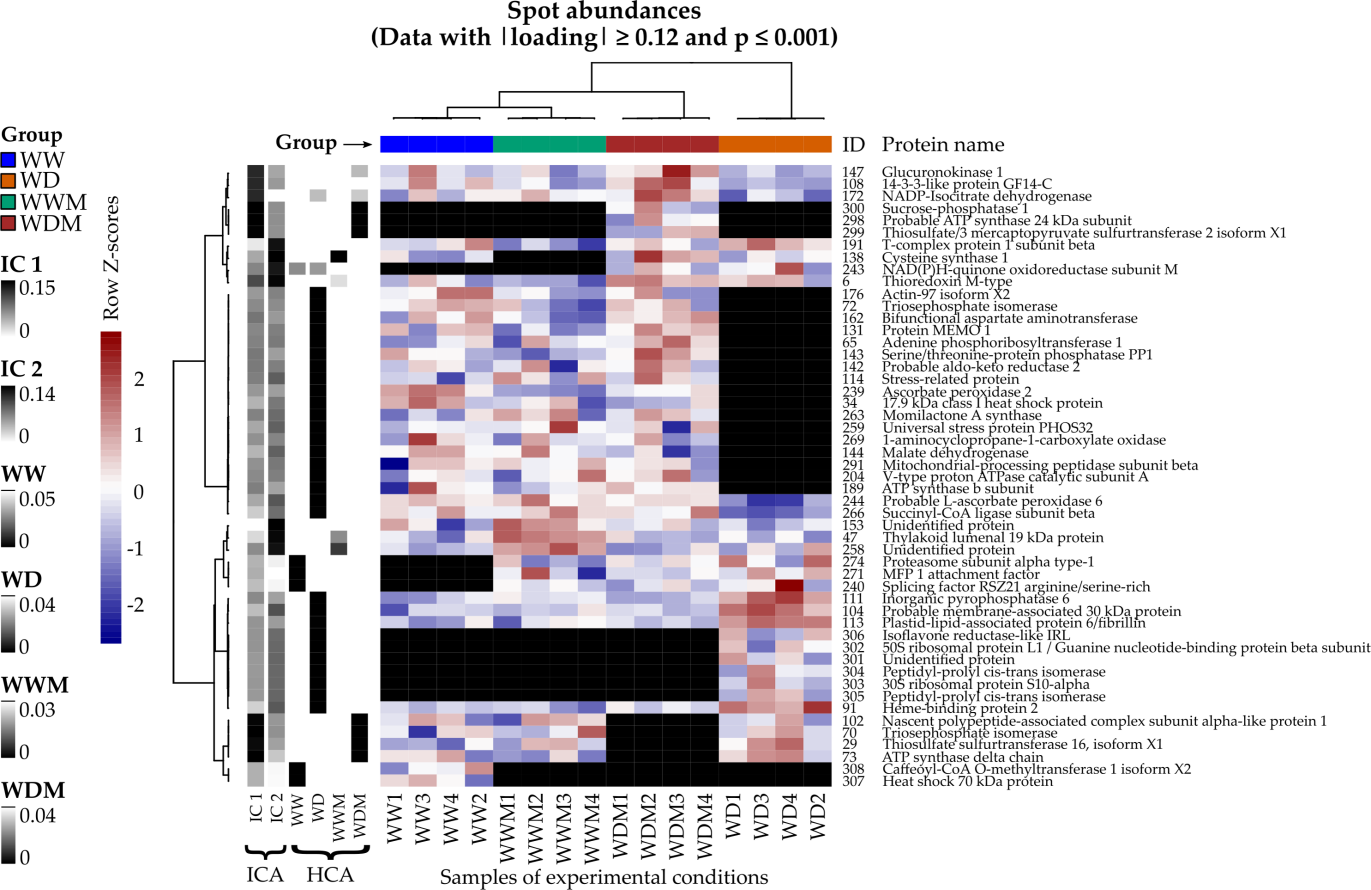
Heat map of differentially accumulated proteins in response to water deficit in mycorrhizal and nonmycorrhizal sorghum plants. Spots and samples were clustered by Ward’s method with the Pearson correlation as distance. Volume spot values are shown as Z scores by row, and each black cell represents an absent protein. Left columns: absolute loadings of the selected spots over the leading ICs and p-values on the detected clusters.

Spots with the highest —loadings— on ICA and the lowest *p*-values on HCA were used to construct a heat map (Fig. 1). From this, ten proteins (spots: 29, 70, 73, 102, 108, 147, 172, 298, 299, 300) were detected that responded to WD and WDM, showing the highest —loadings— on IC1, and seven proteins (spots: 6, 47, 138, 153, 191, 243, 258) responded to the presence of arbuscular mycorrhizal fungi in WWM and WDM, showing the highest —loadings— on IC2. In addition, 41 spots with the lowest *p*-values on HCA correspond to proteins relevant to clusters WW, WD, and WDM (Fig. 1). All the spots selected by ICA and HCA also showed significant differences in their abundances between the groups when an ANOVA was applied.

### Protein identification

**Figure 2:**
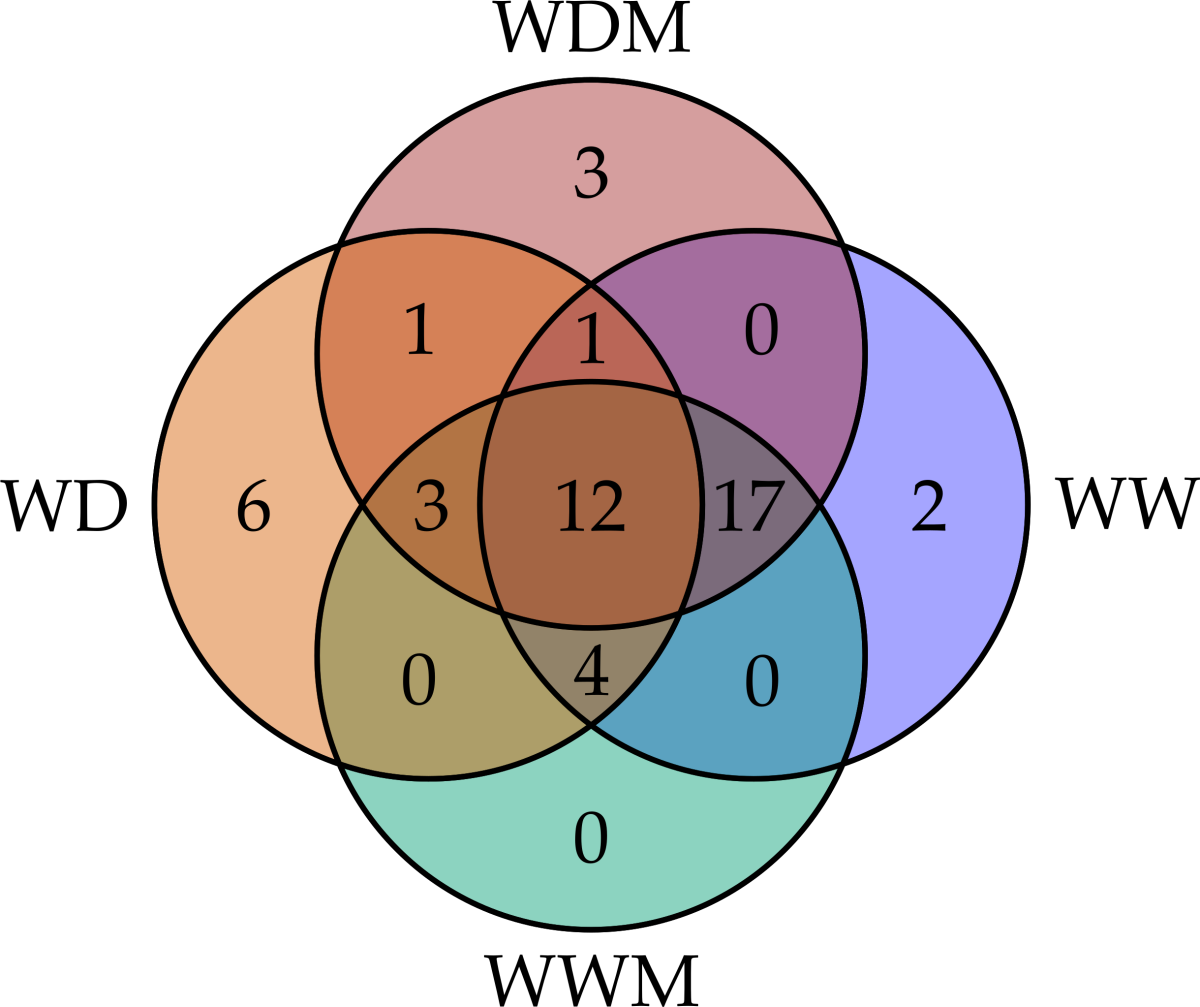
Venn diagram comparing the number of proteins identified for the four experimental conditions. WW and WD represent well-watered and water deficit in nonmycorrhizal plants, while WWM and WDM correspond to well-watered and water deficit mycorrhizal plants.

**Figure 3:**
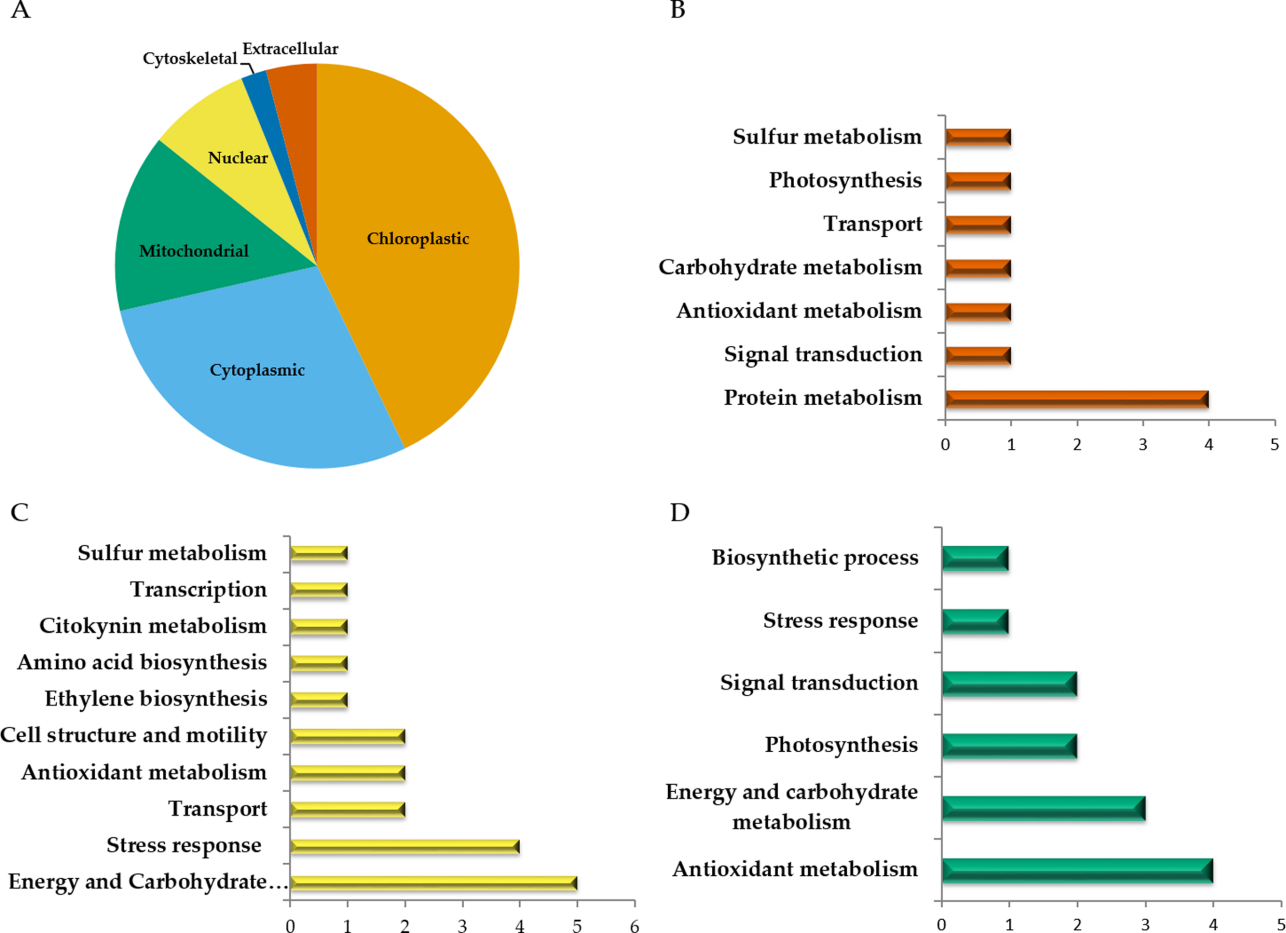
Cellular location and functional classification of differentially accumulated proteins in leaves of mycorrhizal and nonmycorrhizal sorghum plants, under water deficit. (A) Cellular location of all proteins identified. (B) Functional classification of unique proteins in WD. (C) Functional classification of unique proteins in WDM. Functional classification of shared proteins in WD and WDM (D).

Among the 51 proteins with significant changes, we could not identify three proteins. In 28 spots, we identified only one protein per spot, while in the remaining 20 spots, more than one protein per spot was identified. With the exception of spot 302, in which we included the two proteins identified in the same spot, in the remaining spots, we selected the protein with the highest SEQUEST score and similarity to molecular mass and pI of the protein detected in the gel. Finally, forty-nine proteins with significant changes in abundance were identified by HPLC-MS/MS (Tables S1–S4). Additional information about proteins identified is shown in Data S1. Among the differentially accumulated proteins in WD and WDM, we found unique (not found in the contra part) (Tables S1, S2) shared proteins in WD and WDM (Table S3), and others with a variable accumulation pattern in the remaining treatments (only present in WW, only absent in WWM, and absent in WW but present in the remaining treatments) (Table S4). The Venn diagram shows the proteins identified in all experimental conditions (Fig. 2). According to cellular location prediction of all proteins identified, most of them were mainly located in chloroplast (43%), and in a minor proportion in cytoplasm (29%), mitochondria (14%), nuclei (8%), extracellular (4%) and cytoskeleton (2%) (Fig. 3A, Table S5). Based on the functional classification, proteins unique to WD (not found in WDM) were related to protein metabolism, signal transduction, antioxidant metabolism, carbohydrate metabolism, transport, photosynthesis, and sulfur metabolism (Fig. 3B, Table S1). Twenty proteins unique to WDM (not found in WD) were related to energy and carbohydrate metabolism, stress response, transport, antioxidant metabolism, cell structure and motility, ethylene biosynthesis, amino acid biosynthesis, cytokinins metabolism, transcription, and sulfur metabolism (Fig. 3C, Table S2), while thirteen proteins shared in WD and WDM were related to antioxidant metabolism, energy and carbohydrate metabolism, photosynthesis, signal transduction, antioxidant metabolism, stress response and biosynthetic process (Fig. 3D, Table S3).

### Specific proteins accumulated in leaves of WD plants

Sorghum plants under water deficit conditions and in the absence of AM fungi produced six specific proteins absent in any other treatments (Fig. 1, Table S1). Four are related to protein synthesis and folding and included: 50S ribosomal protein L1 (50S-RP-L1) (spot 302a) (sb09g019170), 30S ribosomal protein S10 (30S-RP-S10) (spot 303) (sb01g044040), guanine nucleotide-binding beta subunit-like protein (GNB-β) (spot 302b) (sb09g027690). Two isospecies of peptidyl-prolyl cis-trans isomerase (P-PROL) (spots 304 and 305) (sb09g000350) and an isoflavone reductase-like (ISFR) (spot 306) (sb03g008760). A gene network devised in STRING with a high confidence threshold (0.700) based on *S. bicolor* genes demonstrates a molecular interaction in three proteins, 50S-RP-L1, 30S-RP-S10, and GNB-β. Enrichment analysis with 50 interactors in the first shell demonstrates a molecular interaction in four proteins, except ISFR. Analysis of KEGG pathways revealed that interacting proteins 30S-RP-S10 and 50S-RP-L1 are involved in ribosome biogenesis, while GNB-β (ATARCA homologous in *Arabidopsis thaliana*) is involved in multiple signal transduction pathways, hormone responses, developmental processes, MAPK cascade scaffolding protein in the protease IV and ArgC signaling pathway (Ishida, Takahashi & Nagata, 1993). P-PROL (ROC4, in *A. thaliana*), accelerates the folding of proteins and links light and redox signals to the regulation of cysteine biosynthesis in response to stress (Dominguez-Solis et al., 2008). Analysis of sorghum ISFR, in STRING with medium confidence (0.400) and 50 interactors showed KEGG pathways involved in ribosome biogenesis in eukaryotes and anthocyanin biosynthesis.

### Proteins absent in WDM plants and present in the remaining treatments

Four proteins absent in WDM but present in WW, WD and WWM plants, were: Nascent polypeptide-associated complex subunit alpha-like protein 1, (α-Nasc-AC) (spot 102) (sb07g026160), triosephosphate isomerase (spot 70) (sb03g006130) (TPI), ATP synthase delta chain (ATP-δ) (spot 73) (sb04g027810), thiosulfate sulfurtransferase 16, isoform X1 (TH-ST16) (spot 29) (sb10g030520) (Fig. 1, Table S1). A gene network devised in STRING with a high confidence threshold (0.700) based on *S. bicolor* genes demonstrates no-molecular interaction among these proteins. However, enrichment analysis with 50 interactors demonstrates a molecular interaction in TPI (spot70) (sb03g006130) and ATP-*δ* (spot 73) (sb04g027810). Analysis of KEGG pathways revealed that these two proteins are involved in; respiratory chain, electron transport, mitochondrion, transport, ATP synthesis, and hydrogen ion transport. Individual analyses of α-Nasc-AC (spot 102) (sb07g026160) and TPI (spot70) (sb03g006130), with high confidence (0.700) and enriched with 50 interactors revealed a KEGG pathway involved with ribosome biogenesis for α−Nasc-AC and carbon metabolism, metabolic pathways, glycolysis/gluconeogenesis, carbon fixation in photosynthetic organisms, biosynthesis of secondary metabolites for TPI.

### Specific proteins in WDM plants

Sorghum plants under water deficit conditions with AM-fungi produced three specific proteins absent in any other treatments. They were thiosulfate/3-mercaptopyruvate sulfurtransferase 2 (TH-ST2) (spot 299) (sb08g020860), ATP synthase 24 kDa (ATP-24) (spot 298) (sb04g002620) and sucrose-phosphatase (SPP) (spot 300) (sb04g020180) (Fig. 1, Table S2). A gene network devised in STRING with a medium confidence threshold (0.400) based on *S. bicolor* genes demonstrates that these proteins do not interact among them. Enrichment analysis with 50 interactors revealed a molecular interaction in these three proteins. KEGG pathways analysis showed that they are involved in: metabolic pathways, sulfur metabolism, cysteine and methionine metabolism, starch and sucrose metabolism, oxidative phosphorylation, biosynthesis of amino acids, carbon metabolism and biosynthesis of secondary metabolites. In this network, sucrose-phosphatase interacts with five proteins involved in drought tolerance.

### Proteins absent in WD plants and present in the remaining treatments

Seventeen proteins present in all treatments except in WD plants were found (Fig. 2, Table S2). They include: Mitochondrial-processing peptidase subunit beta (MPP-β) (spot 291) (sb01g043060), V-type proton ATPase catalytic subunit A (V-ATP) (spot 204) (sb04g005040), triosephosphate isomerase (TIM) (spot 72) (sb02g031030), actin (spot 176) (sb09g000750), ATP synthase subunit beta (ATP-β) (spot 189) (sb03g031470), 1-aminocyclopropane-1-carboxylate oxidase (spot 269) (sb03g003550), stress related protein (spot 114) (sb02g042550), probable aldo-keto reductase (spot 142) (sb10g001900), protein MEMO 1 (spot 131) (sb03g046030), bifunctional aspartate aminotransferase (spot 162) (sb09g021360), malate dehydrogenase (MD) (spot 144) (sb03g029570), adenine phosphoribosyltransferase 1 (ADE1) (spot 65) (sb08g019790), ascorbate peroxidase 2 (AP-2) (spot 239) (sb02g044060), momilactone A synthase (spot 263) (sb02g042150), a 17.9 kDa heat shock protein (spot 34) (sb01g040030), universal stress protein (spot 259)  (sb09g004470) and serine/threonine-protein phosphatase (PP1) (spot 143) (sb01g039930) (Fig. 1, Table S2). A gene network devised in STRING with a high confidence threshold (0.700) based on *S. bicolor* genes demonstrates that four proteins interact among them; MPP-β, ATP-β, TIM and MD. Enrichment analysis with 50 interactors with a high confidence (0.700) demonstrates a molecular interaction in six proteins, including V-ATP and AP-2. Analysis of KEGG pathways derived from this interaction are: oxidative phosphorylation, metabolic pathways, carbon metabolism, carbon fixation in photosynthetic organisms, glycolysis/gluconeogenesis, photosynthesis, biosynthesis of secondary metabolites and citrate cycle.

### Shared proteins among treatments

Twelve proteins showed quantitative changes among treatments: plastid-lipid-associated protein 6/fibrillin (spot 113)  (sb05g023220), NADP-Isocitrate dehydrogenase (NADP-ID) (spot 172) (sb06g022050), succinyl-CoA ligase subunit beta (SUCC-CoA) (spot 266) (sb04g026360), L-ascorbate peroxidase 6 (spot 244) (sb08g004880), heme-binding protein 2 (spot 91) (sb03g002090), thioredoxin M-type (THI) (spot 6) (sb08g005260), glucuronokinase 1-like (spot 147) (sb08g000220), 14-3-3-like protein GF14-C (spot 108) (sb07g025680), thylakoid lumenal 19 kDa protein (spot 47) (sb07g027960), probable membrane-associated 30 kDa protein (MEMB) (spot 104) (sb03g042550), putative inorganic pyrophosphatase (I-PYR) (spot 111) (sb04g034340), T-complex protein 1 subunit beta (T-C-β) (spot 191) (sb03g009490) (Fig. 1, Table S3). A gene network devised in STRING with a high confidence threshold (0.700) based on *S. bicolor* genes demonstrates proteins interact between SUCC-CoA (spot 266) (sb04g026360) and NADP-ID (spot 172) (sb06g022050). Enrichment analysis with 50 interactors with a high confidence (0.700) demonstrates a molecular interaction in four proteins; 14-3-3-like protein, THI, SUCC-CoA and NADP-ID. Analysis of KEGG pathways demonstrates that they are involved in: metabolic pathways, oxidative phosphorylation, citrate cycle, carbon metabolism, biosynthesis of secondary metabolites and glycolysis/gluconogenesis.

### Proteins present in WD and WDM plants

NAD(P)H-quinone oxidoreductase subunit M (spot 243) (sb06g023900) was only present in WD and WDM sorghum leaves (Fig. 1, Table S3). Analysis of KEGG pathways demonstrates that this protein is involved in: photosynthesis, metabolic pathways and oxidative phosphorylation.

### Proteins only present in WW plants

Heat shock 70 kDa protein (spot 307) (sb01g017050) and caffeoyl-CoA O-methyltransferase 2 (spot 308) (sb10g004540) were present only in well-watered condition (Fig. 1, Table S4). Gene network analysis demonstrates no molecular interaction between these proteins.

### Proteins present in all treatments, except in WWM plants

Cysteine synthase 1 (spot 138) (sb03g009260) was only absent in WWM (Fig. 1, Table S4). Analysis of the KEGG pathway using 50 interactors in the STRING gene network with a high confidence (0.700) demonstrates its participation in cysteine and methionine metabolism, sulfur metabolism, metabolic pathways, biosynthesis of amino acids, and biosynthesis of secondary metabolites.

### Proteins absent in WW plants

Three proteins present in all treatments, except in WW plants were: splicing factor RSZ21 arginine/serine-rich (spot 240) (sb10g005960), MFP1 attachment factor (spot 271) (sb02g038200) and a proteasome subunit alpha type-1 (spot 274) (sb04g002770) (Fig. 1, Table S4). A gene network devised in STRING with a high confidence threshold (0.700) based on *S. bicolor* genes demonstrates no-protein interaction among them. KEGG pathways analysis with medium confidence (0.400) indicated RSZ21 is involved in, spliceosome and RNA transport, while MFP1 attachment factor with 100 interactors is involved in endocytosis and proteasome subunit alpha type-1, in folding, sorting and protein degradation.

### Gene network derived from proteins altered in WD and WDM plants

**Figure 4:**
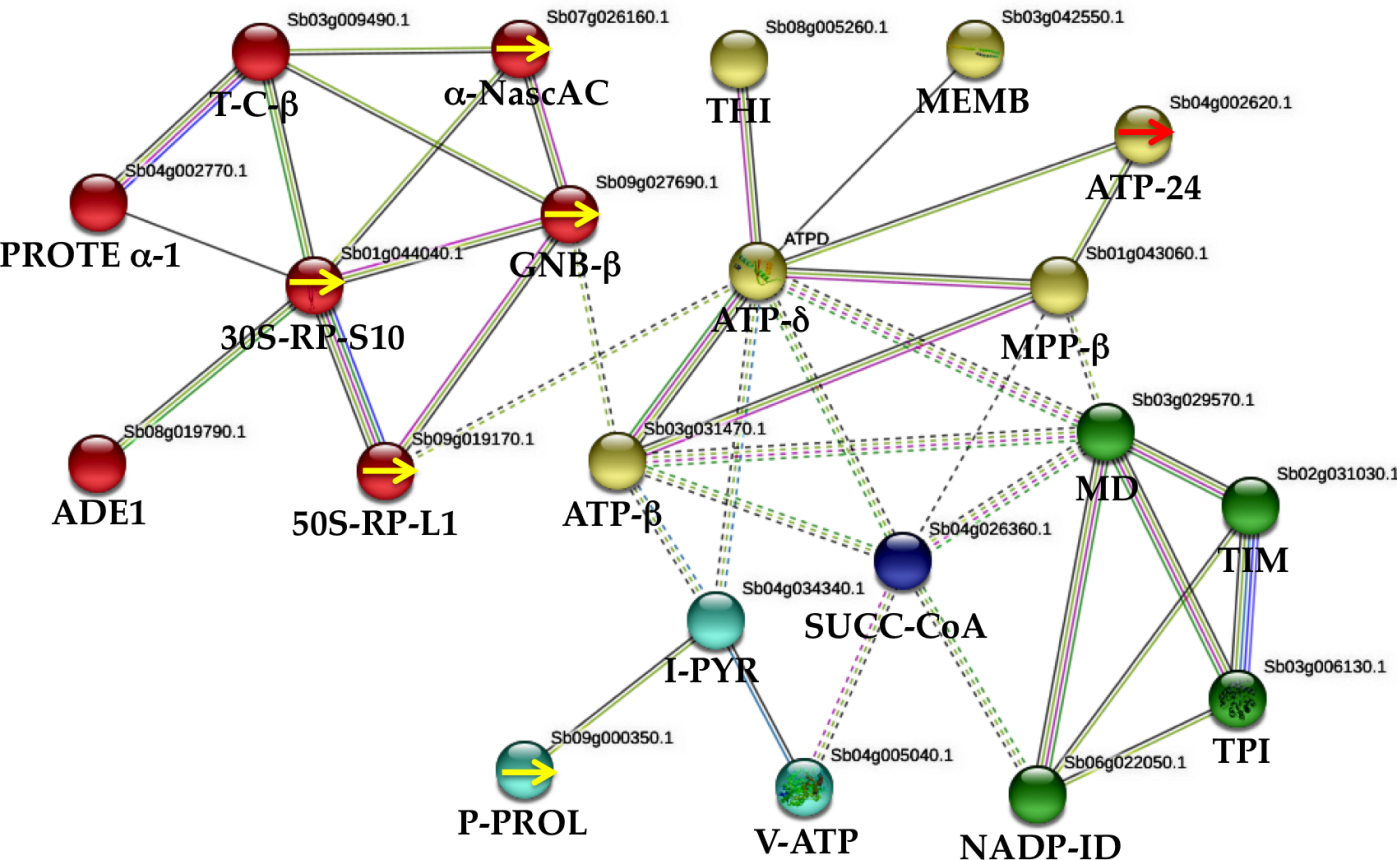
Protein–protein interactions (STRING database, 0.500 confidence) under water deficit in nonmycorrhizal (WD) and mycorrhizal (WDM) S. *bicolor* leaves. The interactions network of twenty one proteins identified, yielded five clusters. Red cluster corresponds to WD plants, while yellow, green and blue clusters to WDM sorghum samples. Yellow arrows indicate specific proteins of WD sorghum plants. Red arrow indicates an specific protein of WDM.

To understand regulatory interactions between sorghum stressed (WD and WDM) and well-watered (WW and WWM), a network derived from STRING-based bioinformatics analysis with (0.500) confidence was performed. Twenty-one proteins out of the 49 identified in this work comprise the interaction network (Fig. 4). The interacting proteins were: four specific proteins from WD 50-RP-L1 (spot 302a) (sb09g019170), 30S-RP-S10 (spot 303) (sb01g044040), (GNB- β) (spot 302b) (sb09g027690) and P-PROL (spots 304 and 305) (sb09g000350). Three proteins, absent in WDM plants and present in the remaining treatments, such as α-Nasc-AC (spot 102) (sb07g026160), TPI (spot70) (sb03g006130) and ATP-δ (spot 73) (sb04g027810). From WDM, one specific protein, ATP-24 (spot 298) (sb04g002620). Six proteins absent in WD plants and present in the remaining treatments; MD (spot 144) (sb03g029570), TIM (spot 72) (sb02g031030), ATP−β (spot 189) (sb03g031470), MPP-β (spot 291) (sb01g043060), ADE1 (spot 65) (sb08g019790), and V-ATP (spot 204) (sb04g005040). Six shared proteins among treatments; T-C-β (spot 191) (sb03g009490), I-PYR (spot 111) (sb04g034340), THI (spot 6) (sb08g005260), MEMB (spot 104) (sb03g042550), NADP-ID (spot 172) (sb06g022050), and SUCC-CoA (spot 266) (sb04g026360). One protein absent only in WW plants; PROTE α-1 (spot 274) (sb04g002770). MCL clustering with seven inflation parameters demonstrates five clusters (Fig. 4). Enrichment analysis with 50 interactors with a (0.500) confidence formed four clusters based on KEGG pathways. One cluster related to WD was mainly involved in ribosome biogenesis (four proteins), and secondarily with oxidative phosphorylation and carbon metabolism, while in WDM, two clusters emerged; one involved with oxidative phosphorylation, photosynthesis, metabolic pathways and the second one with carbon metabolism, carbon fixation in photosynthetic organisms, glycolysis/gluconeogenesis, citrate cycle, and biosynthesis of secondary metabolites. The fourth cluster is a proteasome interacting with the above clusters (Fig. 4).

## Discussion

Sorghum is a drought crop model that depends on the action and interaction of morphological, physiological, biochemical, and genetic mechanisms that condition these characters (Mitra, 2001). It has been largely demonstrated that arbuscular mycorrhizal (AM) improves the growth of host plants by promoting nutrient and water uptake to alleviate abiotic stresses, such as drought (Baum, El-Tohamy & Gruda, 2015; Zhao et al., 2015; Bowles, Jackson & Cavagnaro, 2018). To understand the molecular mechanism involved in drought tolerance, physiological and proteomic analyses were made in sorghum plants alone or in combination with a consortium of six species of arbuscular mycorrhizal (AM) fungi. Our results, together with the protein–protein interactions analysis, revealed significant differences in response to water deficit in WD and WDM sorghum plants.

### Physiological responses of WD and WDM sorghum plants

In our work, a mild water deficit treatment was used, which did not affect mycorrhizal colonization (Table 1). In addition, sorghum inoculation with the arbuscular mycorrhizal consortium increased growth and biomass in both WWM and WDM plants (Table 2). This increase in growth and biomass was similar to previously reported in sorghum with another mycorrhizal inoculum (Sun, Shi & Ding, 2017; Nakmee, Techapinyawat & Ngamprasit, 2016).

A significant reduction in soil water potential in WD and WDM sorghum plants was observed (Table 3). However, WDM plants were able to adjust this stress and maintained their leaves water potential very similar to WW sorghum plants as it was also found in other studies (Augé, Toler & Saxton, 2015). Our results are in agreement with the 23–29% increase in stomatal conductance reported in mycorrhizal plants under drought by Augé, Toler & Saxton (2015), after a meta-analysis of 460 studies. In the present work, the rate of CO_2_ assimilation was higher in WWM than WW sorghum plants, and these values decreased in response to water deficit as it has been previously reported in other mycorrhizal plants (Porcel & Ruiz-Lozano, 2004; Birhane et al., 2012). It was also noticed that the CO_2_ assimilation was higher in WDM than WD sorghum plants (Table 3). Recently, it was reported that water deficit alters photosynthetic machinery in plants, but mycorrhizal plants maintain a suitable CO_2_ assimilation rate to be productive (Santander et al., 2017). Our results are consistent with the suggestion that the mycorrhizal fungi association enhances photosynthesis in plants, under normal and water deficit conditions (Table 3).

### Network interaction predictions based on differential expression in WD sorghum plants

In sorghum plants exposed to water deficit (WD), protein synthesis and turnover coupled with a protein involved in multiple signal transduction pathways was found in this investigation. These results were expected since proteomic investigation revealed that 16% of proteins are attributed to protein synthesis, which is a fundamental metabolic process for plants to cope with drought stress (Wang et al., 2016). In this work, PPI analysis of the altered proteins retrieved from the STRING software with (0.500) confidence, showed interaction of a specific protein in WD plants, with four proteins (Fig. 4). GNB-β involved in multiple signal transduction pathways, which modulate stress responses. It is homologous to ATARCA gene found in *Arabidopsis thaliana*, which is the major component of the Rack1 regulatory proteins that play multiple roles in signal transduction pathways, such as hormone responses (auxins, Wang, Zhang & Wu, 2016; ABA, Guo et al., 2011), H_2_O_2_ (Anand et al., 2019) and developmental processes (Chen et al., 2006). GNB-β interacts with α-Nasc-AC, a protein found in WD, WW, and WM. α-Nasc-AC is part of a heterodimeric complex (alpha and beta units) which binds to nascent proteins at the ribosomes and regulates the co-translational protein import and the targeting of translating ribosomes with mitochondria (Lesnik et al., 2014). Zhang et al. (2010) demonstrates that a protein elicitor purified from *Botrytis cinerea* was highly homologous to α-Nasc-AC. Treatment of wheat seedlings with this protein promoted root growth and induce drought tolerance, while the application in tomato plant increased the activity of defensive enzymes, inducing disease resistance against *B. cinerea* (Zhang et al., 2010). GNB-β also interacts with two specific proteins in WD plants; 50S-RP-L1 and 30-RP-S10 (Fig. 4). Ribosomal proteins have an important role in forming and stabilizing the ribosomal complex and mediating protein translation. They also participate in the regulation of gene expression and DNA repair. Most of the ribosomal genes and proteins have been found up-regulated in response to drought (Moin et al., 2017). In addition, ribosome biogenesis is one of the major energy-consuming cellular processes and is under tight regulation in response to environmental signals (Martin, Soulard & Hall, 2004). GNB-β also interacts with one protein involved in protein folding, T-C-β (T-complex protein) (Fig. 4). Following the network, 30S-RP-S10 interacts with PROTE α-1 and ADE1, a protein involved in recycling adenine (Fig. 4). PROTE α-1 has an ATP-dependent proteolytic activity, negatively regulates thiol biosynthesis and arsenic tolerance. Within the same network, 50S-RP-L1 interacts with a protein found in WD, WW, and WM; ATP synthase delta chain (ATP- *δ*) a chloroplastic F(1)F(0), essential for photosynthesis (Fig. 4). ATP synthase delta chain interacts with a protein found in all treatments; membrane-associated protein VIPP1, a “very important protein in plastids”, is required for plastid vesicle formation and thylakoid membrane biogenesis. The fourth and fifth specific proteins in WD plants were the two isoforms of peptidyl-prolyl cis-trans isomerase. P-PROL interacts with a protein found in all treatments, a putative inorganic pyrophosphatase (I-PYR) that interacts with the ATP synthase delta chain (Fig. 4). P-PROL is homologous to ROC4 in *A. thaliana*. This enzyme catalyzes the reversible conversion of peptidyl-prolyl bond from cis to trans configuration, which is a rate-limiting step in the folding of proteins. It also links light and redox signals to the regulation of cysteine biosynthesis in response to stress. P-PROL prevents Rubisco damage in rice under drought stress (Ji et al., 2012). It is involved in signal transduction through the modulation of Ca^2+^ dependent phosphatase activity, and its activity has been demonstrated in *S. bicolor* seedlings under water stress (Sharma et al., 2003). Overexpression of the P-PROL gene (OsCYP18-2) enhanced drought tolerance in rice and *A. thaliana* plants, altering the expression and RNA splicing patterns by interacting with SKIP (Lee et al., 2015). *A. thaliana* transgenic plants overexpressing the P-PROL gene (*FKBP12*) of an alpine haircap moss were tolerant to multiple types of abiotic stress, including heat, ABA, drought and salt stress (Alavilli et al., 2018). Isoflavone reductase was the sixth specific protein found in WD plants; its accumulation correlated with drought stress imposition and protects cells from oxidative stress by a glutathione-independent mechanism (Babiychuk et al., 1995). Proteomic analysis of soybean subjected to water deficit identified two IFR, with contrasting changes in response to water deficit (Alam et al., 2010).

Interestingly, our PPI network resulted in WD sorghum plants showed coincidences with the expression profile of drought-responsive genes selected by QTL and characterized by Abou-Elwafa & Shehzad (2018). Gene of GNB-β (spot 302b) (sb09g027690) found in our work, interacts with genes characterized by QTL, such as mitogen-activated protein-kinase (sb10g003810), 40S ribosomal protein S12 (sb02g006810), 40S ribosomal protein S8 (sb04g028530) and elongation factor EFTS (sb02g041940) (Fig. S3). This may suggest that the set of proteins we found in our analysis is part of a common metabolic pathway, which can represent an evolutionarily conserved mechanism in sorghum plants that promotes the maintenance of clusters involved in drought tolerance like those found in the research of Abou-Elwafa & Shehzad (2018).

### Network interaction predictions based on differential expression in WDM sorghum plants

In WDM sorghum plants, a different response was revealed. PPI analysis revealed that ATP-24kD, one specific protein in WDM sorghum plants, interacts with ATP-β and MPP-β (Fig. 4). ATP-24kDa (homologous to MGP1 in *A. thaliana*) is a copper, cobalt, and zinc ion binding, mitochondrial ATP synthase. In *A. thaliana*, MGP1 is essential for pollen formation during dehydration at the later developmental stage of pollen grains (drought stress) (Li et al., 2010). ATP-24kD has been implicated in male sterility in sorghum (Sane, Nath & Sane, 1994), in cell adaptation to drought stress in wheat (Pastore et al., 2007) and in cytoplasmic male sterility in cotton (Kong et al., 2019). Overexpression of a mitochondrial ATP synthase small subunit (AtMtATP6) gene in yeast and arabidopsis increased tolerance to drought, salinity, oxidative, and cold stresses (Zhang, Liu & Takano, 2008). In our network, ATP-24kD interacts with MPP-β (Fig. 4), this protein cleaves the mitochondrial targeting peptide from precursor proteins, to enter to the matrix through Tim17:23 complex. It was demonstrated that reduced levels of ATP in the Tim21 mutant (*sd3*) resulted in a dwarf phenotype (Hamasaki et al., 2012). Changes in mitochondrial protein abundance upon stress, are associated with the increase of broken peptides (ATP synthase subunits) caused by high proteolytic activity (Sweetlove et al., 2002). At water stress conditions, it was demonstrated that a decrease of ATP synthase reduces photosynthesis due to the inhibition of ribulose biphosphate carboxylase synthesis (Tezara et al., 1999). We also found that MPP-β interacts with ATP-β and MD (Fig. 4). MD is essential to shuttle reductants out from the mitochondria to support the photorespiratory flux. This enzyme catalyzes a reversible NAD-dependent dehydrogenase reaction involved in central metabolism and redox homeostasis. MD interacts with TIM and two proteins present in all treatments; SUCC-CoA ligase and NADP-ID (Fig. 4). TIM is a chloroplastic triosephosphate isomerase, essential for activation of the entire energy-producing pathway to maintain homeostasis in stressed cells. Genes involved in ATP synthesis are activated in culture cells of rice grown with high (20%) sucrose content, inducing the production of glycolytic enzymes to overcome stress conditions. Under these conditions, TIM, glyceraldehyde phosphate dehydrogenase, and phosphoglycerate kinase were up-regulated (Umeda et al., 1994). Triosephosphate isomerase has been largely documented in drought tolerance plants, like wheat (Caruso et al., 2009; Ge et al., 2012; Cheng et al., 2016), rice (Gorantla et al., 2007; Sharma et al., 2012), chickpea (Khanna et al., 2014), common-bean (Zadražnik et al., 2013), coffee (Menezes-Silva et al., 2017) and sorghum (Dhawi, Datta & Ramakrishna, 2017).

Proteomic studies have revealed that nearly 20% of drought-responsive proteins are involved in carbohydrate and energy metabolism (glycolysis, TCA cycle, electron transport chain and ATP synthesis (Wang et al., 2016). Sucrose-phosphatase, a specific protein in WDM sorghum plants, catalyzes the final step of sucrose synthesis. It interacts in the network using low confidence (0.125) with TIM. The most prominent role of sucrose is as a sugar transport molecule. In addition, sucrose initiates signaling pathways to alter gene expression for physiological adaptation (Wind, Smeekens & Hanson, 2010). Another specific protein found in WDM sorghum plants was TH-ST2; this protein did not interact in this network. It catalyzes the transfer of a sulfur ion form to cyanide or to other thiol compounds. Sulfurtransferases have been involved in cyanide detoxification in the cytoplasm and in the protection of mitochondrial cytochrome c oxidase (Nagahara, Ito & Minami, 1999), as well as in heavy metal stress (Louie, Kondor & Dewitt, 2003), and resistance against fungal pathogens. TH-ST2 interacts with cysteine synthase 1 (CY-S), a protein present in all treatments, except in WWM plants. To alleviate oxidative stress, glutathione (GSH) functions as a direct antioxidant, and cysteine is essential for GSH synthesis. Energy metabolism is one of the basic metabolic pathways in plants, and AMF can promote photosynthesis and respiration in plants (Porcel et al., 2015; Romero-Munar et al., 2017). In this study, the increase of various proteins related to photosynthesis, oxidative phosphorylation (ATP-24kD, ATP-*β*, V-ATP, I-PYR), citrate cycle (MD, SUCC-CoA, NADP-ID) and carbon metabolism (TIM) was coincident with the higher levels of CO_2_ assimilation and stomatal conductance observed in WDM compared to WD plants (Table 3).

In an extensive transcriptomic analysis of sorghum under field conditions, a drop in fungal colonization and gene expression critical to arbuscular symbiosis was found after prolonged drought (Varoquaux et al., 2019). In our work, a mild water deficit treatment was used, which did not affect mycorrhizal colonization (Table 1).

## Conclusions

Our proteomic analysis indicates that mycorrhizal and nonmycorrhizal sorghum plants use different molecular mechanisms to face water deficit stress.

In nonmycorrhizal sorghum plants, the main activated KEGG pathways were ribosome biogenesis, protein biosynthesis, folding and carbon metabolism. According to the literature these pathways play a crucial role in adaptation to water deficit.

However, in mycorrhizal plants, the main activated KEGG pathways were energy, carbon and sulfur metabolism, oxidative phosphorylation, glycolysis/gluconeogenesis, and photosynthesis.

A synergetic interaction using PPI showed four clusters among specific proteins accumulated in WD and WDM sorghum plants.

##  Supplemental Information

10.7717/peerj.8991/supp-1 Figure S1 Representative two-dimensional electrophoresis profiles of proteins in sorghum leaves underdifferent water deficit treatments Proteins (1 mg) extracted with the phenol-ammonium method were separated by IEF in 18 cm IPG strips, pH range 4–7] followed by 12.5% sodium dodecyl sulfate polyacrylamide gel electrophoresis (SDS-PAGE) and subsequently stained with Coomassie Brilliant Blue G-250. The molecular mass (Mm) in kilodaltons (kDa) and pH are indicated on the right and at the bottom of the gels, respectively. WW and WD indicate well-watered and water deficit nonmycorrhizal plants, while WWM and WDM correspond to well-watered and water deficit mycorrhizal plants, respectively.

10.7717/peerj.8991/supp-2 Figure S2 Multivariate analysis of protein spots detected by 2DE in mycorrhizal (WWM and WDM) and nonmycorrizal (WW and WD) sorghum plants Four experimental groups are shown: WW, well-watered nonmycorrhizal plants; WD, water deficit nonmycorrhizal plants; WWM, well-watered mycorrhizal plants; WDM, water deficit mycorrhizal plants. (A) Independent component analysis of the PCA scores (PCA-ICA): projections with 95% confidence ellipses are shown in the space spanned by the two leading independent components. IC1 mainly represents the effects related to water stress, while IC2 shows the effects of inoculation with arbuscular mycorrhizal fungi. (B) Hierarchical clustering analysis of the PCA scores (PCA-HCA) based on Euclidean distance and Ward’s clustering, validated by bootstrap analysis (AU: approximately unbiased probability).

10.7717/peerj.8991/supp-3 Figure S3 Protein-protein interactions network resulted in WD and WDM sorghum plants showed coincidences with the expression profile of drought tolerance genes selected by QTL (Abou-Elwafaand Shehzad 2018) Black arrows indicate genes selected by QTL and interacting with GNB- b.

10.7717/peerj.8991/supp-4 Tabla S1 Proteins identified unique to WD sorghum plants * indicates more than one protein was identified. Accumulation values are (% relative volume spot) ^1∕3^. Bars represent the mean of four biologically independent measurements ±standard error.
1 and 2 refers to well-watered (WW) and water deficit (WD) nonmycorrhizal plants, respectively; while 3 and 4 to well-watered (WWM) and water deficit (WDM) mycorrhizal plants, respectively.

10.7717/peerj.8991/supp-5 Tabla S2 Proteins identified unique to WDM sorghum plants * indicates more than one protein was identified. Accumulation values are (% relative volume spot) ^1∕3^. Bars represent the mean of four biologically independent measurements ±standard error.
1 and 2 refers to well-watered (WW) and water deficit (WD) nonmycorrhizal plants, respectively; while 3 and 4 to well-watered (WWM) and water deficit (WDM) mycorrhizal plants, respectively.

10.7717/peerj.8991/supp-6 Tabla S3 Proteins identified shared in WD and WDM sorghum plants * indicates more than one protein was identified. Accumulation values are (% relative volume spot) ^1∕3^. Bars represent the mean of four biologically independent measurements ±standard error.
1 and 2 refers to well-watered (WW) and water deficit (WD) nonmycorrhizal plants, respectively; while 3 and 4 to well-watered (WWM) and water deficit (WDM) mycorrhizal plants, respectively.

10.7717/peerj.8991/supp-7 Tabla S4 Proteins identified with different patterns of accumulation in sorghum plants * indicates more than one protein was identified. Accumulation values are (% relative volume spot) ^1∕3^. Bars represent the mean of four biologically independent measurements ±standard error.
1 and 2 refers to well-watered (WW) and water deficit (WD) nonmycorrhizal plants, respectively; while 3 and 4 to well-watered (WWM) and water deficit (WDM) mycorrhizal plants, respectively.

10.7717/peerj.8991/supp-8 Tabla S5 Cellular location of proteins identified in leaves from mycorrhizal and nonmycorrhizal sorghum plants at water deficit conditions *Gene accession number in the most likely orthologous plant species, when they did not match with sorghum.Cellular location was predicted using the public program WolfPsort ( https://www.genscript.com/wolf-psort.html).Chlo: chloroplast, Cysk: Cytoskeletal, Cyto: Cytoplasmic, Ext: Extracellular, Nucl: Nuclear, Mito: Mitochondrial

10.7717/peerj.8991/supp-9 Data_1 Proteins identified in response to water deficit in mycorrhizal and nonmycorrhizal sorghum plants

## Abbreviations

50S-RP-L1: 50S ribosomal protein L1
30S-RP-S10: 30S ribosomal protein S10
GN-β: Guanine nucleotide-binding beta subunit
ISFR: Isoflavone reductase-like
α-Nasc-AC: Nascent polypeptide-associated complex subunit alpha-like
ATP-δ: ATP synthase delta chain
TPI: Triosephosphate isomerase
MD: Malate dehydrogenase
TIM: Triosephosphate isomerase
ATP-β: ATP subunit beta
AP-2: Ascorbate peroxidase 2
ATP-24: ATP synthase 24 kDa
SPP: Sucrose-phosphatase
MPP-β: Mitochondrial-processing peptidase subunit beta
PROTE α 1: Proteasome subunit alpha type-1
T-C-β: T-complex protein 1 subunit beta
NADP-ID: NADP-Isocitrate dehydrogenase
SUCC-CoA: SUCC-CoA ligase subunit beta
I-PYR: Putative inorganic pyrophosphatase
THI: Thioredoxin M-type
MEMB: Probable membrane-associated 30 kDa protein
V-ATP: V-type proton ATPase catalytic subunit A
CY-S: Cysteine synthase 1
ADE1: Adenine phosphoribosyltransferase 1
TH-ST2: Thiosulfate/3-mercaptopyruvate sulfurtransferas 2
HSP: Heat shock 70 kDa protein
P-PROL: Peptidyl-prolyl cis-trans isomerase
TH-ST16: Thiosulfurtransferase 16, isoform X1

## Additional Information and Declarations

### Data Availability

The following information was supplied regarding data availability: Data is available at Zenodo: Olalde-Portugal, Victor; Cabrera-Ponce, José Luis; Gastelum-Arellanez, Argel; Guerrero-Rangel, Armando; Winkler, Robert, & Valdés-Rodríguez, Silvia. (2020). Proteomic analysis reveals different molecular mechanisms to face water deficit in mycorrhizal and nonmycorrhizal sorghum plants [Data set]. Zenodo. http://doi.org/10.5281/zenodo.3604404.
