# Supplementary figures and images for: Proteomic analysis and interactions network in leaves of mycorrhizal and nonmycorrhizal sorghum plants under water deficit
Source: PeerJ. 2020 Apr 23;8:e8991. doi: 10.7717/peerj.8991 (PMC7183753; doi:10.7717/peerj.8991)

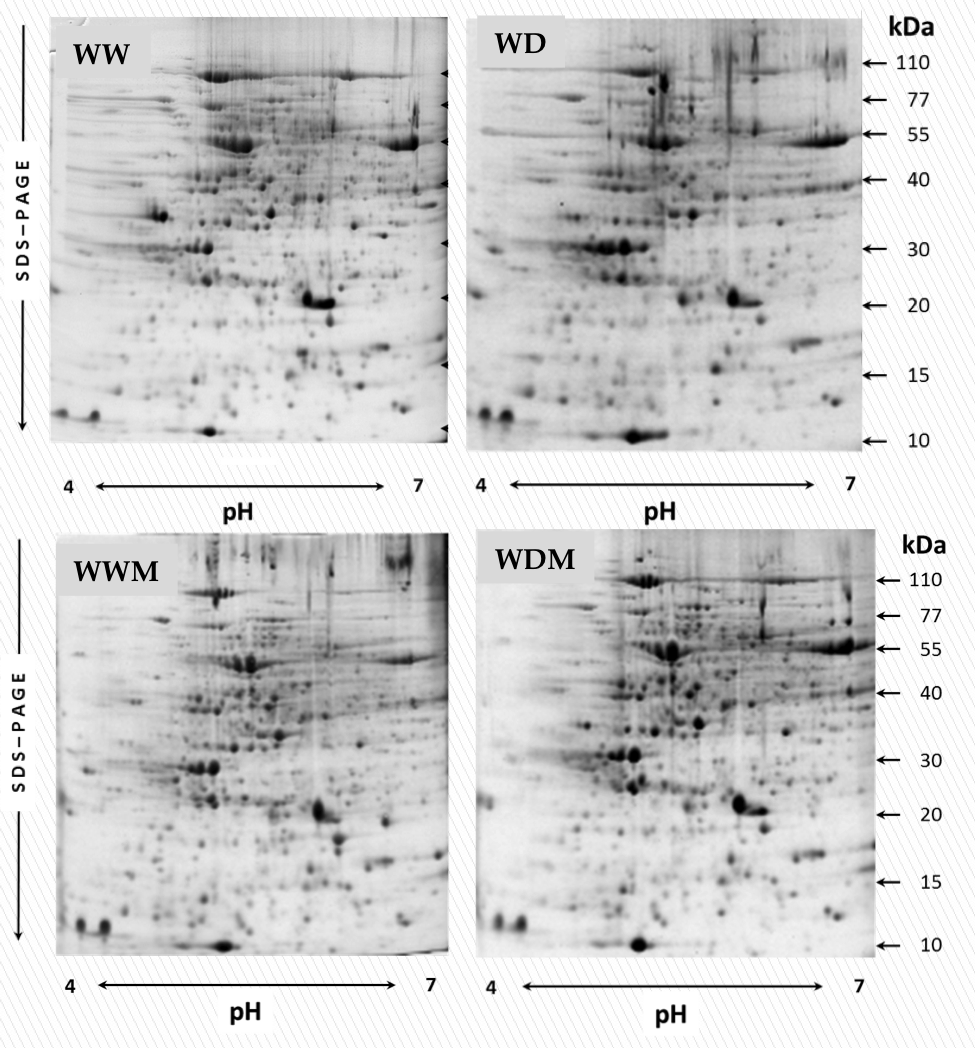

Supplement: Figure S1 — Proteins (1 mg) extracted with the phenol-ammonium method were separated by IEF in 18 cm IPG strips, pH range 4–7] followed by 12.5% sodium dodecyl sulfate polyacrylamide gel electrophoresis (SDS-PAGE) and subsequently stained with Coomassie Brilliant Blue G-250. The molecular mass (Mm) in kilodaltons (kDa) and pH are indicated on the right and at the bottom of the gels, respectively. WW and WD indicate well-watered and water deficit nonmycorrhizal plants, while WWM and WDM correspond to well-watered and water deficit mycorrhizal plants, respectively. [file peerj-08-8991-s001.png]

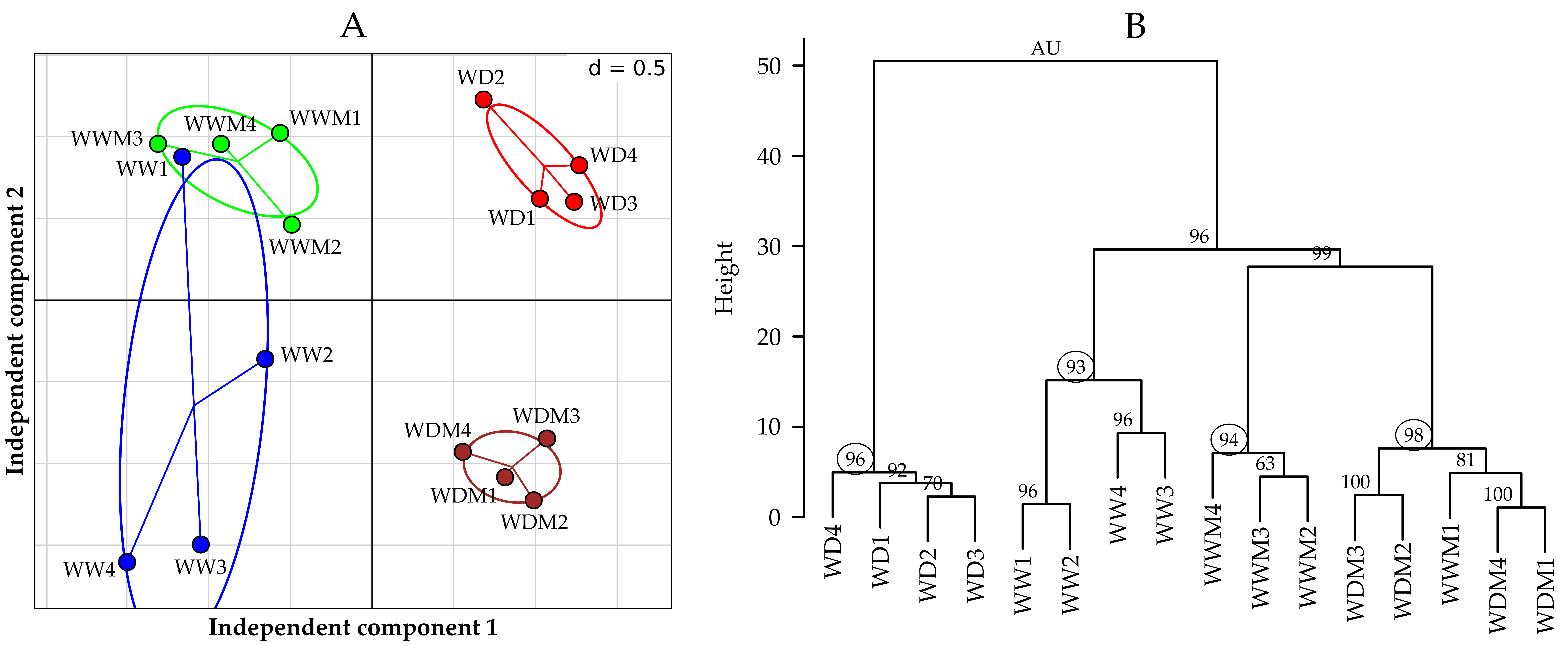

Supplement: Figure S2 — Four experimental groups are shown: WW, well-watered nonmycorrhizal plants; WD, water deficit nonmycorrhizal plants; WWM, well-watered mycorrhizal plants; WDM, water deficit mycorrhizal plants. (A) Independent component analysis of the PCA scores (PCA-ICA): projections with 95% confidence ellipses are shown in the space spanned by the two leading independent components. IC1 mainly represents the effects related to water stress, while IC2 shows the effects of inoculation with arbuscular mycorrhizal fungi. (B) Hierarchical clustering analysis of the PCA scores (PCA-HCA) based on Euclidean distance and Ward’s clustering, validated by bootstrap analysis (AU: approximately unbiased probability). [file peerj-08-8991-s002.png]

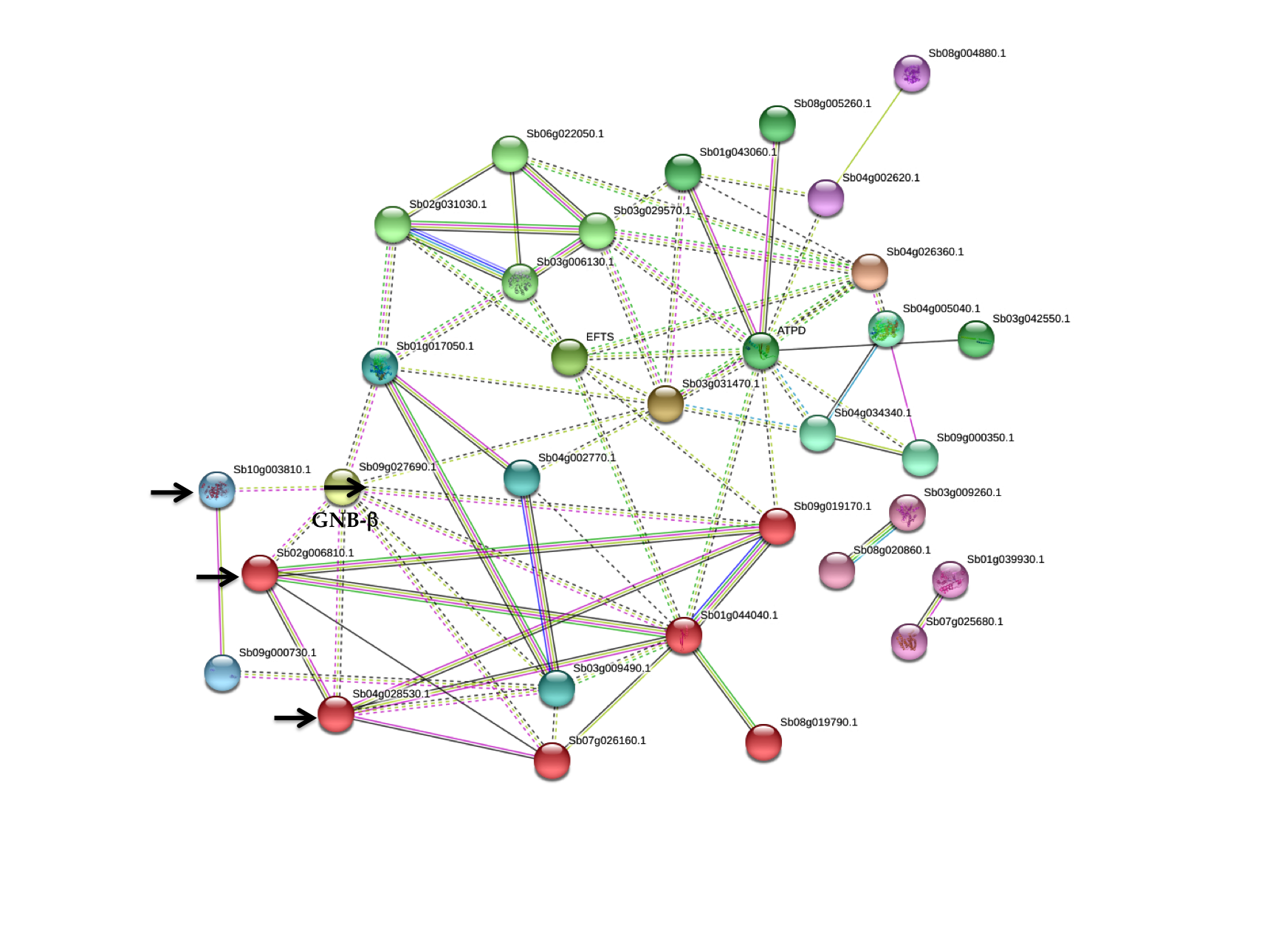

Supplement: Figure S3 — Black arrows indicate genes selected by QTL and interacting with GNB- b. [file peerj-08-8991-s003.png]
