# Supplementary material for: Proteomic analysis and interactions network in leaves of mycorrhizal and nonmycorrhizal sorghum plants under water deficit
Source: PeerJ. 2020 Apr 23;8:e8991. doi: 10.7717/peerj.8991 (PMC7183753; doi:10.7717/peerj.8991)
Supplement: Tabla S1 — * indicates more than one protein was identified. Accumulation values are (% relative volume spot) 1∕3. Bars represent the mean of four biologically independent measurements ±standard error. 1 and 2 refers to well-watered (WW) and water deficit (WD) nonmycorrhizal plants, respectively; while 3 and 4 to well-watered (WWM) and water deficit (WDM) mycorrhizal plants, respectively. [file peerj-08-8991-s004.docx]

| **Spot** | **SORBIDRAFT** | **Protein name** | **Protein functional category** | **Accumulation level** |
| --- | --- | --- | --- | --- |
|  |  |  |  | **1 2 3 4** |
| *302a | sb09g019170 | 50S ribosomal protein L1 | Protein metabolism | 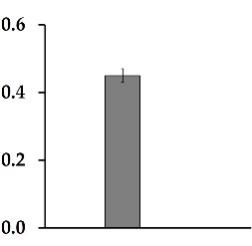 |
| 303 | sb01g044040 | 30S ribosomal protein S10-α  *Zea Mays*  (GI: PWZ06987) | Protein metabolism | 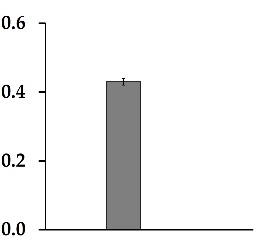 |
| *302b | sb09g027690 | Guanine nucleotide-binding protein β-subunit | Signal transduction | 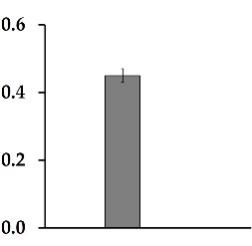 |
| 304 | sb09g000350 | Peptidyl-prolyl cis-trans isomerase | Protein metabolism | 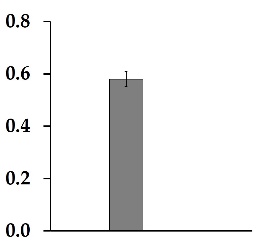 |
| 305 | sb09g000350 | Peptidyl-prolyl cis-trans isomerase | Protein metabolism | 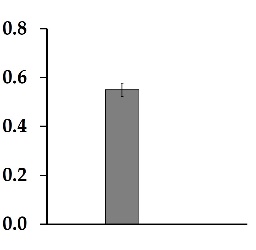 |
| 306 | sb03g008760 | Isoflavone reductase-like IRL | Antioxidant metabolism | 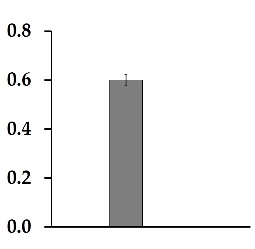 |
| 102 | sb07g026160 | Nascent polypeptide-associated complex subunit α-like protein 1 | Transport | 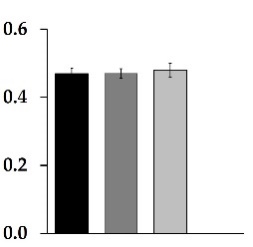 |
| 70 | sb03g006130 | Triosephosphate isomerase | Carbohydrate metabolism | 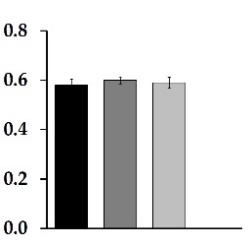 |
| 73 | sb04g027810 | ATP synthase delta chain | Photosynthesis | 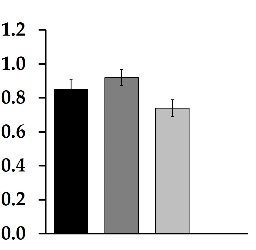 |
| 29 | sb10g030520 | Thiosulfate sulfurtransferase 16, isoform X1 | Sulfur metabolism | 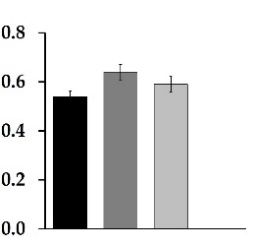 |
