# Supplementary material for: Proteomic analysis and interactions network in leaves of mycorrhizal and nonmycorrhizal sorghum plants under water deficit
Source: PeerJ. 2020 Apr 23;8:e8991. doi: 10.7717/peerj.8991 (PMC7183753; doi:10.7717/peerj.8991)
Supplement: Tabla S2 — * indicates more than one protein was identified. Accumulation values are (% relative volume spot) 1∕3. Bars represent the mean of four biologically independent measurements ±standard error. 1 and 2 refers to well-watered (WW) and water deficit (WD) nonmycorrhizal plants, respectively; while 3 and 4 to well-watered (WWM) and water deficit (WDM) mycorrhizal plants, respectively. [file peerj-08-8991-s005.docx]

| **Spot** | **SORBIDRAFT** | **Protein name** | **Protein functional category** | **Accumulation level** |
| --- | --- | --- | --- | --- |
|  |  |  |  | **1 2 3 4** |
| *299 | sb08g020860 | Thiosulfate/3 mercaptopyruvate sulfurtransferase 2 isoform X1 | Sulfur metabolism | 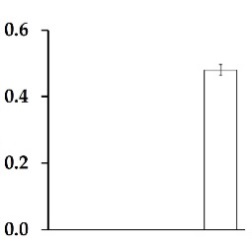 |
| *298 | sb04g002620 | Probable ATP synthase 24 kDa subunit | Energy metabolism | 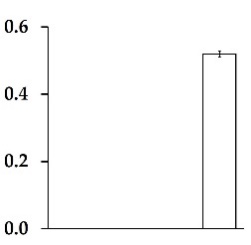 |
| *300 | sb04g020180 | Sucrose-phosphatase 1 | Carbohydrate metabolism | 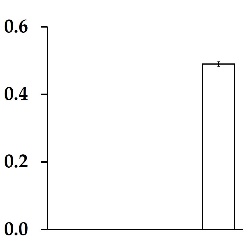 |
| 291 | sb01g043060 | Mitochondrial-processing peptidase subunit β | Transport | 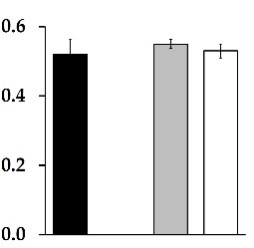 |
| 204 | sb04g005040 | V-type proton ATPase catalytic subunit A | Transport | 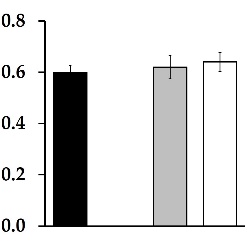 |
| 72 | sb02g031030 | Triosephosphate isomerase | Carbohydrate metabolism | 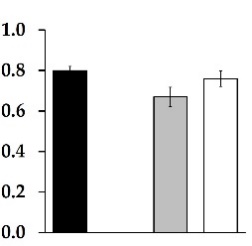 |
| 176 | sb09g000730 | Actin-97 isoform X2,  *Zea mays*  (GI: 103629276) | Cell structure | 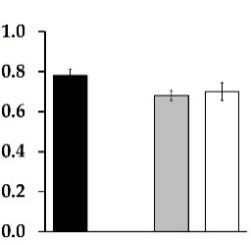 |
| *189 | sb03g031470 | ATP synthase β subunit | Energy metabolism | 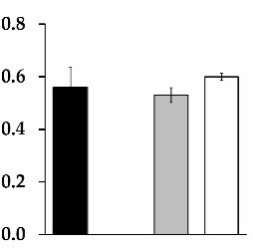 |
| *269 | sb03g003550 | 1aminocyclopropane-1-carboxylate oxidase | Ethylene biosynthesis | 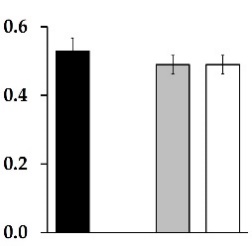 |
| *114 | sb02g042550 | Stress-related protein | Stress response | 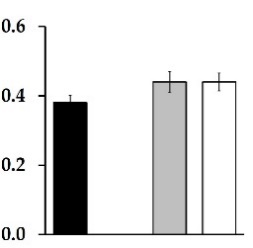 |
| *142 | sb10g001900 | Probable aldo-keto reductase 2 | Stress response | 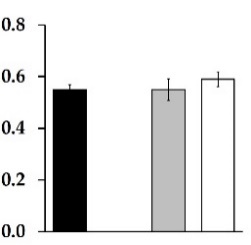 |
| *131 | sb03g046030 | Protein MEMO 1 | Cell motility | 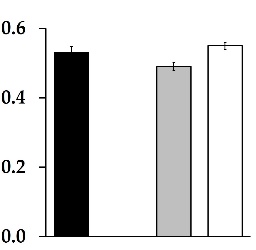 |
| *162 | sb09g021360 | Bifunctional aspartate aminotransferase and glutamate/aspartate-prephenate aminotransferase | Amino acid Biosynthesis | 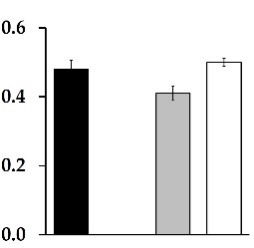 |
| 144 | sb03g029570 | Malate dehydrogenase | Carbohydrate metabolism | 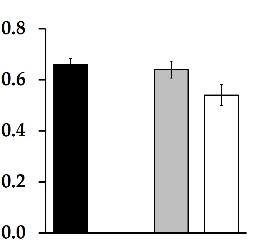 |
| *65 | sb08g019790 | Adenine phosphoribosyl transferase 1 | Cytokinin metabolism | 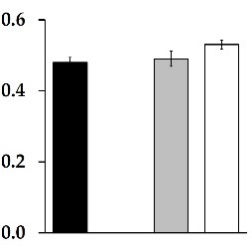 |
| 239 | sb02g044060 | Ascorbate peroxidase 2 | Antioxidant metabolism | 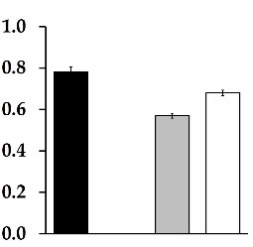 |
| 263 | sb02g042150 | Momilactone A synthase | Antioxidant metabolism | 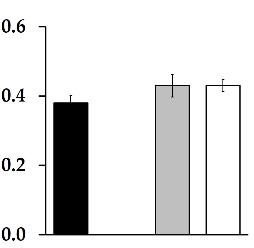 |
| 34 | sb01g040030 | 17.9 kDa class I heat shock protein | Stress response | 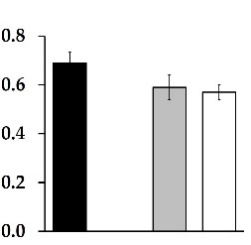 |
| 259 | sb09g004470 | Universal stress protein PHOS32 | Stress response | 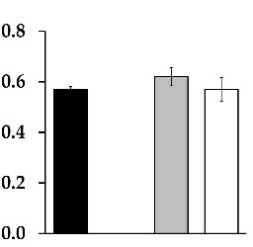 |
| *143 | sb01g039930 | Serine/threonine-protein phosphatase PP1 isoform X1,  *Zea mays*  (GI: 103634267) | Transcription | 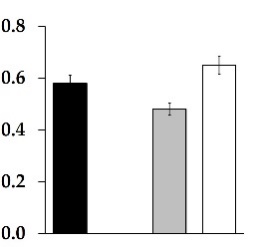 |
