# Supplementary material for: Proteomic analysis and interactions network in leaves of mycorrhizal and nonmycorrhizal sorghum plants under water deficit
Source: PeerJ. 2020 Apr 23;8:e8991. doi: 10.7717/peerj.8991 (PMC7183753; doi:10.7717/peerj.8991)
Supplement: Tabla S3 — * indicates more than one protein was identified. Accumulation values are (% relative volume spot) 1∕3. Bars represent the mean of four biologically independent measurements ±standard error. 1 and 2 refers to well-watered (WW) and water deficit (WD) nonmycorrhizal plants, respectively; while 3 and 4 to well-watered (WWM) and water deficit (WDM) mycorrhizal plants, respectively. [file peerj-08-8991-s006.docx]

| **Spot** | **SORBIDRAFT** | **Protein name** | **Protein functional category** | **Accumulation level** |
| --- | --- | --- | --- | --- |
|  |  |  |  | **1 2 3 4** |
| 113 | sb05g023220 | Plastid-lipid-associated protein 6/fibrillin | Stress response | 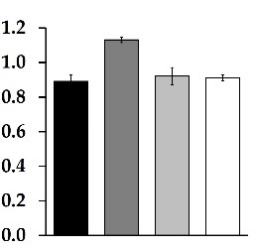 |
| *172 | sb06g022050 | NADP-Isocitrate dehydrogenase | Carbohydrate metabolism | 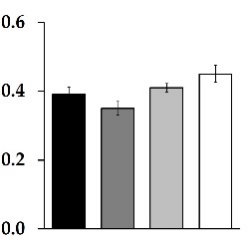 |
| *266 | sb04g026360 | Succinyl-CoA ligase subunit beta | Carbohydrate metabolism | 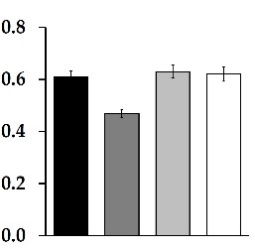 |
| 244 | sb08g004880 | Probable L-ascorbate peroxidase 6 | Antioxidant metabolism | 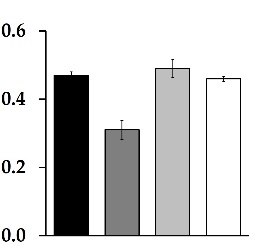 |
| 91 | sb03g002090 | Heme-binding  protein 2 | Antioxidant metabolism | 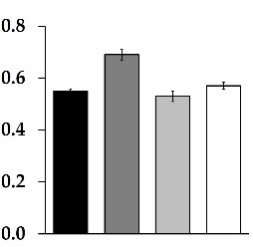 |
| 6 | sb08g005260 | Thioredoxin M-type | Antioxidant metabolism | 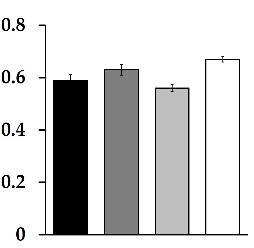 |
| *147 | sb08g000220 | Glucuronokinase 1 | Biosynthetic Process | 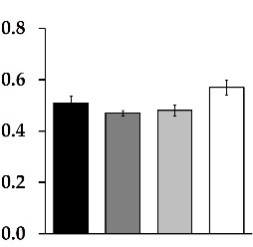 |
| *108 | sb07g025680 | 14-3-3-like protein GF14-C | Signal  transduction | 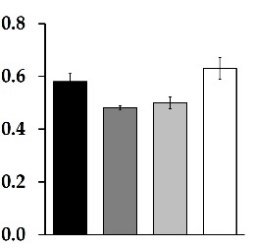 |
| 47 | sb07g027960 | Thylakoid lumenal 19 kDa protein | Photosynthesis | 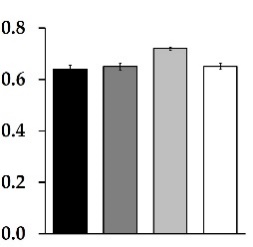 |
| 104 | sb03g042550 | Probable membrane-associated 30 kDa protein | Photosynthesis | 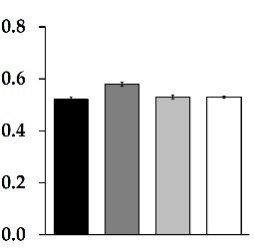 |
| 111 | sb04g034340 | Inorganic pyrophosphatase 6 | Energy metabolism | 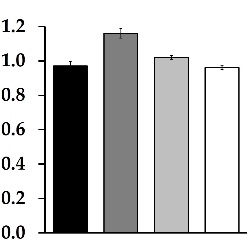 |
| *191 | sb03g00949*0* | T-complex protein 1 subunit β | Signal transduction | 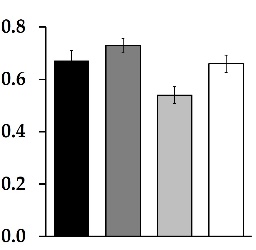 |
| 243 | sb06g023900 | NAD(P)H-quinone oxidoreductase subunit M | Antioxidant metabolism | 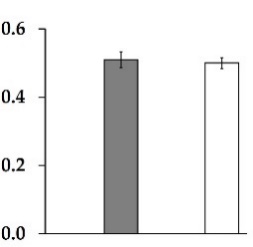 |
