# Supplementary material for: Proteomic analysis and interactions network in leaves of mycorrhizal and nonmycorrhizal sorghum plants under water deficit
Source: PeerJ. 2020 Apr 23;8:e8991. doi: 10.7717/peerj.8991 (PMC7183753; doi:10.7717/peerj.8991)
Supplement: Tabla S4 — * indicates more than one protein was identified. Accumulation values are (% relative volume spot) 1∕3. Bars represent the mean of four biologically independent measurements ±standard error. 1 and 2 refers to well-watered (WW) and water deficit (WD) nonmycorrhizal plants, respectively; while 3 and 4 to well-watered (WWM) and water deficit (WDM) mycorrhizal plants, respectively. [file peerj-08-8991-s007.docx]

| **Spot** | **SORBIDRAFT** | **Protein name** | **Protein functional category** | **Accumulation level** |
| --- | --- | --- | --- | --- |
|  |  |  |  | **1 2 3 4** |
| *307 | sb01g017050 | Heat shock 70 kDa protein  *Panicum miliaceum,*  (GI: RNL19808.1) | Stress response | 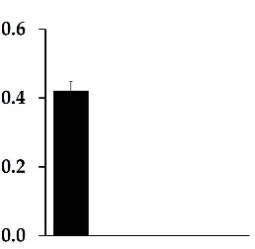 |
| *308 | sb10g004540 | Caffeoyl-CoA O-methyltransferase 1 isoform X2 | Biosynthesis  Flavonoids | 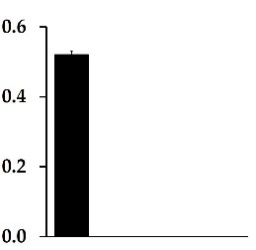 |
| 138 | sb03g009260 | Cysteine synthase 1 | Sulfur metabolism | 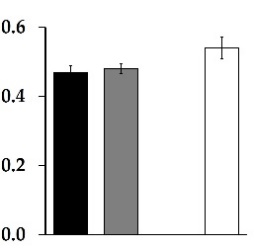 |
| 240 | sb10g005960 | Splicing factor RSZ21 arginine/serine-rich | RNA processing | 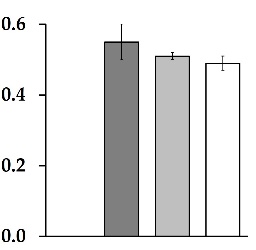 |
| 271 | sb02g038200 | MFP 1 attachment factor | Unknown function | 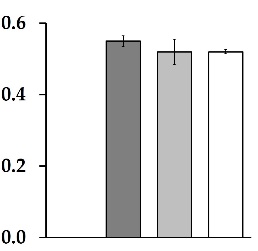 |
| *274 | sb04g002770 | Proteasome subunit α type-1 | Protein metabolism | 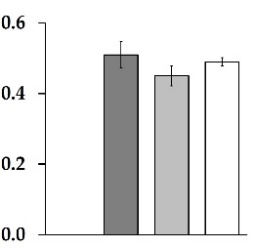 |
