# Supplementary material for: Proteomic analysis and interactions network in leaves of mycorrhizal and nonmycorrhizal sorghum plants under water deficit
Source: PeerJ. 2020 Apr 23;8:e8991. doi: 10.7717/peerj.8991 (PMC7183753; doi:10.7717/peerj.8991)
Supplement: Tabla S5 — *Gene accession number in the most likely orthologous plant species, when they did not match with sorghum.Cellular location was predicted using the public program WolfPsort ( https://www.genscript.com/wolf-psort.html).Chlo: chloroplast, Cysk: Cytoskeletal, Cyto: Cytoplasmic, Ext: Extracellular, Nucl: Nuclear, Mito: Mitochondrial [file peerj-08-8991-s008.docx]

| **Spot number** | **SORBIDRAFT** | **Protein name** | **Cellular location** |
| --- | --- | --- | --- |
| 302a | sb09g019170 | 50S ribosomal protein L1 | Chlo |
| 303 | sb01g044040 | 30S ribosomal protein S10-α  *Zea Mays*  (*GI: PWZ06987) | Chlo |
| 302b | sb09g027690 | Guanine nucleotide-binding protein β-subunit | Cyto |
| 304 | sb09g000350 | Peptidyl-prolyl cis-trans isomerase | Chlo |
| 305 | sb09g000350 | Peptidyl-prolyl cis-trans isomerase | Chlo |
| 306 | sb03g008760 | Isoflavone reductase-like IRL | Cyto |
| 102 | sb07g026160 | Nascent polypeptide-associated complex subunit α-like protein 1 | Nucl |
| 70 | sb03g006130 | Triosephosphate isomerase | Cyto |
| 73 | sb04g027810 | ATP synthase delta chain | Chlo |
| 29 | sb10g030520 | Thiosulfate sulfurtransferase 16, isoform X1 | Chlo |
| 299 | sb08g020860 | Thiosulfate/3 mercaptopyruvate sulfurtransferase 2 isoform X1 | Cyto |
| 298 | sb04g002620 | Probable ATP synthase 24 kDa subunit | Mito |
| 300 | sb04g020180 | Sucrose-phosphatase 1 | Chlo |
| 291 | sb01g043060 | Mitochondrial-processing peptidase subunit β | Mito |
| 204 | sb04g005040 | V-type proton ATPase catalytic subunit A | Chlo |
| 72 | sb02g031030 | Triosephosphate isomerase | Chlo |
| 176 | sb09g000730 | Actin-97 isoform X2,  *Zea mays* (*GI: 103629276) | Cysk |
| 189 | sb03g031470 | ATP synthase β subunit | Cyto |
| 269 | sb03g003550 | 1-aminocyclopropane-1-carboxylate oxidase | Chlo |
| 114 | sb02g042550 | Stress-related protein | Cyto |
| 142 | sb10g001900 | Probable aldo-keto reductase 2 | Cyto |
| 131 | sb03g046030 | Protein MEMO 1 | Mito |
| 162 | sb09g021360 | Bifunctional aspartate aminotransferase and glutamate/aspartate-prephenate aminotransferase | Chlo |
| 144 | sb03g029570 | Malate dehydrogenase | Mito |
| 65 | sb08g019790 | Adenine phosphoribosyl transferase | Chlo |
| 239 | sb02g044060 | Ascorbate peroxidase 2 | Mito |
| 263 | sb02g042150 | Momilactone A synthase | Cyto |
| 34 | sb01g040030 | 17.9 kDa class I heat shock protein | Cyto |
| 259 | sb09g004470 | Universal stress protein PHOS32 | Cyto |
| 143 | sb01g039930 | Serine/threonine-protein phosphatase PP1 isoform X1  *Zea mays* (*GI: 103634267) | Cyto |
| 113 | sb05g023220 | Plastid-lipid-associated protein 6/fibrillin | Chlo |
| 172 | sb06g022050 | NADP-Isocitrate dehydrogenase | Chlo |
| 266 | sb04g026360 | Succinyl-CoA ligase subunit beta | Mito |
| 244 | sb08g004880 | Probable L-ascorbate peroxidase 6 | Extr |
| 91 | sb03g002090 | Heme-binding protein 2 | Extr |
| 6 | sb08g005260 | Thioredoxin M-type | Chlo |
| 147 | sb08g000220 | Glucuronokinase 1 | Cyto |
| 108 | sb07g025680 | 14-3-3-like protein GF14-C | Nucl |
| 47 | sb07g027960 | Thylakoid lumenal 19 kDa protein | Chlo |
| 104 | sb03g042550 | Probable membrane-associated 30 kDa protein | Chlo |
| 111 | sb04g034340 | Inorganic pyrophosphatase 6 | Chlo |
| 191 | sb03g00949*0* | T-complex protein 1 subunit β | Cyto |
| 243 | sb06g023900 | NAD(P)H-quinone oxidoreductase subunit M | Chlo |
| 307 | sb01g017050 | Heat shock 70 kDa protein  *Panicum miliaceum,*  *(GI: RNL19808.1) | Mito |
| 308 | sb10g004540 | Caffeoyl-CoA O-methyltransferase 1 isoform X2 | Cyto |
| 138 | sb03g009260 | Cysteine synthase 1 | Chlo |
| 240 | sb10g005960 | Splicing factor RSZ21 arginine/serine-rich | Nucl |
| 271 | sb02g038200 | MFP 1 attachment factor | Nucl |
| 274 | sb04g002770 | Proteasome subunit α type-1 | Chlo |
